# Symptom-specific gut microbial and metabolic profiles in ADHD reveal SCFA deficiency as a Key pathogenic mechanism

**DOI:** 10.1080/19490976.2025.2537755

**Published:** 2025-07-27

**Authors:** Xinyue Wang, Ning Wang, Teng Gao, Yunfan Zhang, Zhao Fu, Yilu Zhao, Yujingwen Huang, Xiangyu Zheng, Xuping Gao, Lin Lu, Li Yang

**Affiliations:** a National Clinical Research Center for Mental Disorders, Peking University Sixth Hospital, Peking University Institute of Mental Health, Beijing, China; b Department of Medicine, Peking University, Beijing, China

**Keywords:** Attention deficit hyperactivity disorder, metabolites, microbiota-gut-brain axis, fecal microbiota transplantation

## Abstract

Previous evidence links gut microbiota to attention-deficit/hyperactivity disorder (ADHD) through the gut-brain axis. However, the specific microbiota contributing to symptoms remain unclear. To characterize the gut microbial profile related to different symptoms and explore the mediation mechanism between microbiota alterations and the core ADHD symptoms, we conducted shotgun metagenomic sequencing and fecal metabolomics analysis on 94 ADHD patients and 94 age- and gender-matched controls. Microbial characteristics of three subgroups exhibiting different ADHD core symptom presentations were analyzed. We developed a metabolic model and conducted causal mediation analyses to examine how metabolites connect the microbiota to the symptoms. Fecal microbiota transplantation in mice was employed to validate the findings. The redundancy analysis identified ADHD symptoms as environmental gradients and explained the changes in beta diversity (F = 1.345, pFDR = 0.015). Greater gut microbial alterations were observed in combined presentations (ADHD-C). Several beneficial bacteria involved in short-chain fatty acid synthesis were found to be downregulated, with Lactobacillus sanfranciscensis notably linked to all three core symptoms (p.adj = 1.04E–13; p.adj = 5.07E–07; p.adj = 2.61E–05). Various taxa, functional pathways, and metabolites associated with specific ADHD symptom domains were identified. Imidazoleacetic acid partially mediated the effects between Lactobacillus sanfranciscensis and inattention (p = 0.012). In mice subjected to feces from ADHD patients with a low abundance of Lactobacillus sanfranciscensis, treatment with this strain greatly improved both hyperactivity (t = 2.665, p = 0.0237) and inattention (t = 2.389, p = 0.0380), while acetate supplementation only alleviated inattention (t = 2.362, p = 0.0398). Our findings suggest that different ADHD symptoms were related to common and different gut microbiota and metabolites. Fecal microbiota transplantation in mice validated the hypothesis that gut microbial composition affects ADHD symptoms through metabolic alterations. This study provides more insight into the mechanisms underlying metabolic disturbances in ADHD and elucidates the role of gut microbiota in these processes.

## 1. Introduction

Attention-deficit/hyperactivity disorder (ADHD) is one of the most commonly diagnosed psychiatric conditions in children and adolescents, with a global prevalence of 3–5%.^1^ ADHD is a complex condition characterized by significant heterogeneity, evident in variability in clinical presentations and developmental trajectories, diverse psychiatric comorbidities, and extensive structural and functional brain abnormalities.^2,3^ The three core symptoms of ADHD share common yet distinct mechanisms, involving prefrontal-striatal circuits dysfunction, catecholamine dysregulation, cortical maturation delays, and other factors.^4^

Although ADHD is highly heritable,^5,6^ increasing evidence indicates that environmental factors also play a significant role in its development. Various environmental factors, including perinatal factors,^7,8^ postnatal social-emotional factors,^9,10^ and dietary or micronutrient intake,^11,12^ may influence the severity of ADHD symptoms.

Through the microbiota-gut-brain axis (MGBA), the effects of the gut microbiota on neurodevelopment have gained more recognition recently. The gut microbiota is commonly considered to regulate neurodevelopment through three pathways – the immune pathway, the neuronal pathway, and the endocrine/systemic pathway, with overlaps and interaction in between.^13^ Some studies have revealed the role of the MGBA in neurodevelopmental disorders.^14,15^ It has been demonstrated that early disturbances to the developing gut microbiota may affect neurodevelopment, potentially leading to adverse mental health outcomes later.^16^ One study reported that in children who developed neurodevelopmental disorders, specific gut microbial populations decreased at certain developmental stages.^17^

Research has shown that many ADHD-related risk factors also influence the microbiota, such as delivery method, gestational age, type of feeding, maternal health, and early life stressors.^18,19^ Clinical evidence indicates that early antibiotic exposure may be associated with an increased risk of ADHD,^20,21^ and cesarean delivery, which affects early gut microbiota colonization, is considered a risk factor for ADHD.^22^ Some interventional studies on ADHD further support the role of the MGBA in pathogenesis. For instance, probiotics such as *Bifidobacterium* and ketogenic diets have been shown to alleviate ADHD-like behavioral symptoms in rat models by modulating the gut microbiota.^23,24^ Early probiotic treatment has also been associated with improved long-term outcomes.^25^ These findings suggest thorough assessment and intervention of the gut microbiota in ADHD patients.

Previous studies utilizing 16S rRNA gene amplicon or shotgun metagenomic sequencing have preliminarily identified differences in gut microbiota composition between ADHD and controls.^26–33^ However, findings regarding ADHD have been inconsistent across studies, and the key structure and composition of the microbiota in ADHD remain unclear. Additionally, the specific mechanisms by which gut microbiota contribute to different pathology and behavioral presentation are still not well understood. In this context, we aim to further characterize the gut microbial profile in ADHD, investigating whether alterations in gut microbiota and its functions may contribute to inattention, hyperactivity, and impulsivity symptoms. Additionally, we hope to explore potential targeted therapeutic avenues for ADHD by the regulation of gut microbiota.

## 2. Methods and material

### 2.1. Participants

One hundred and eighty-eight participants aged 6–16 years were recruited from the Child and Adolescent Mental Health Center at Peking University Sixth Hospital, Yan’an Third People’s Hospital, and local schools. Of these, 94 were ADHD patients and 94 were age- and gender-matched healthy controls. The initial diagnoses of patients were made by senior psychiatrists using the DSM-5 criteria,^34^ which were subsequently confirmed by trained investigators conducting semi-structural interviews using the Chinese version of the KSADS-PL.^35^ The number of participants in the three subgroups was as follows: 56 ADHD-I patients (predominantly inattentive presentation, group IA), 9 ADHD-HI patients (predominantly hyperactivity-impulsivity presentation, group HA) and 29 ADHD-C patients (combined presentation, group C).

Patients with ADHD and healthy controls were excluded when they met the following exclusion criteria: (1) history of treatment with any medication for ADHD; (2) use of psychoactive drugs, antibiotics, probiotics, or traditional Chinese medicine within 1 month prior to sample collection; and (3) special dietary preferences (such as vegan or ketogenic diets) or significant changes in diet within the previous month. Participants were excluded if they had comorbid psychiatric, neurological, gastrointestinal, or metabolic conditions. Additionally, individuals with IQs below 70 according to Raven’s Standard Progressive Matrices^36^ were excluded.

Following the guidelines outlined in the Declaration of Helsinki, the research was granted authorization by the Ethics Committee of Peking University Sixth Hospital (Approval No. 2021–79). Informed consent was obtained from legal guardians, while children aged 8 years or older signed the informed consent on their own.

### 2.2. Clinical assessment

#### 2.2.1. Attention-deficit/Hyperactivity disorder rating scale (ADHD-RS)

ADHD symptoms were measured using the Attention-Deficit/Hyperactivity Disorder Rating Scale,^37^ which consists of 18 items separated into two dimensions: inattention and hyperactivity-impulsivity. The first nine items pertain to inattention symptoms, while the remaining nine items pertain to hyperactivity-impulsivity symptoms. The frequency of behaviors is assessed using a 4-point scale: 0 points for ‘never,’ 1 point for ‘occasionally,’ 2 points for ‘often,’ and 3 points for ‘always.’ Based on the severity of symptoms in two dimensions, patients can be classified into ADHD-I (predominantly inattentive presentation), ADHD-HI (predominantly hyperactivity-impulsivity presentation), and ADHD-C (combined presentation).

#### 2.2.2. Children behavior Checklist (CBCL)

The Children Behavior Checklist (CBCL), a widely recognized children behavior assessment tool,^38^ is mostly used to assess behavioral and emotional problems in children and adolescents. Raw scores of eight domains (Withdrawn, Somatic Complaints, Anxious/Depressed, Social Problems, Thought Problems, Attention Problems, Delinquent Behavior, and Aggressive Behavior) were calculated. A raw score representing the severity of the problem is obtained by adding the items on each CBCL subscale, with higher scores corresponding to a more serious problem.

#### 2.2.3. Conners parental symptom questionnaire (PSQ)

The Conners Parental Symptom Questionnaire (PSQ) is completed by the child’s primary caregiver based on the child’s daily behavior.^39^ The 48 items on the questionnaire are, respectively, scored on a range of 0 to 3. Hyperactivity-impulsivity, hyperactivity index, conduct issues, learning issues, psychosomatic issues, and anxiety are its six factors. Higher scores on any factor indicate a more serious behavioral problem in that area.

#### 2.2.4. Dietary assessment

Food frequency questionnaires (FFQ) are an effective instrument for assessing typical dietary intake and its relationship with health and disease outcomes.^40^ A semi-quantitative FFQ that collected information on average habitual dietary intake was used to evaluate dietary information. The average frequency and portion size of each food’s consumption over the previous 12 months were asked of the participants. Frequencies and portions of each individual food item were converted to average daily intake for each participant. Nutrient values were calculated using the Chinese Food Composition Table.^41^ By multiplying the frequency of consumption of each food by its nutrient content and adding up all of the foods, we were able to determine the average daily nutrient and total energy intakes (TEI). After being adjusted by dividing by each person’s total energy intake, the energy, carbohydrate, protein, fat, fiber, cholesterol, fatty acid, and several amino acids were measured and quantified. The results were then standardized into Z-scores.^42,43^ We used SPSS (v26.0) to conduct principal component analysis (PCA) with maximal rotation of variance in order to address multicollinearity among nutrients.^44^ In later analyses, the principal components (PC) with a minimum eigenvalue of 1.0 in the principal component analysis were kept as covariates.

### 2.3. Sample collection and DNA extraction

Standardized procedures were followed for fecal sample collection, after which the samples were frozen at −80°C. Microbial DNA was extracted from 200 mg fecal samples from 188 participants using the QIAamp PowerFecal Pro DNA Kit. The DNA concentration was measured using a microplate reader, and its integrity was assessed by agarose gel electrophoresis.

### 2.4. Metagenomic analysis

Samples were sequenced on the MGISEQ 2000 platform by Beijing Genomics Institute using PE150 mode with an insert size of 300–400 bp. Prior to bioinformatic analysis, SOAPnuke (v.2.2.1)^45^ was used to trim all raw data, and raw reads containing adapter sequences (more than 15 bases aligned to the connector sequence), low-quality reads (lower Q-score 20 rate more than 50%), and ambiguous bases (N base rate more than 0.1%) were filtered out. Bowtie2 (v.2.4.4) software^46^ was then employed to map the data to the host genome (GRCh37/hg19), identified, and eliminated host genome sequences to minimize interference in subsequent analyses.

High-quality short reads from each DNA sample were assembled into contigs using a multiple k-mer size strategy with MEGAHIT software^47^ and sequence assemblies were subjected to length-based filtration using a 300-bp cutoff threshold. Genes were predicted across the obtained contigs using MetaGeneMark software. CD-HIT software^48^ was employed to cluster sequences and reduce redundancy, cluster classification was performed based on sequence similarity criteria (pairwise identity ≥95%, alignment coverage ≥90%). Salmon software was utilized for quantification to construct the gene abundance matrix. To determine the phylogenetic origin and functional annotations of the metagenomic clusters, Diamond BLASTP was used to search against the KEGG and Swiss-Prot databases. Taxonomic annotation was assigned based on the Kraken2 LCA algorithm with a default setting,^49^ using the Unified Human Gastrointestinal Genome (UHGG) database. The default settings of Bracken2 software were utilized to produce taxonomic abundance profiles. For per-feature tests, we first performed quality control filtering for taxonomic and functional features before including them in the subsequent analyses. To be qualified for downstream analyses, a taxonomic feature or pathway had to be found in at least one-third of samples and have a minimum total relative abundance of 0.01%.

### 2.5. Metabolomics analysis

All participants were initially assigned sequential subject identifiers upon recruitment. After completing fecal sample collection, all samples were randomly reordered using the sample() function in R software (version 4.2.0), and new identification numbers (ADHD: A001-A094; Controls: H001-H094) were assigned accordingly. Metabolomics profiling was performed on the first 31 ADHD patients (A001-A031) and 31 controls (H001-H031) based on the ascending order of these IDs. The 20 μL sample and 20 μL standard were mixed with HM400 releasing agent for extraction. Following centrifugation at 18,000 g for 10 min at 4°C, the supernatant was collected for LC-MS/MS analysis.

Metabolite separation and quantification were carried out using a Waters ACQUITY UPLC I-Class Plus (Waters, USA) in conjunction with a QTRAP 6500+ high-sensitivity mass spectrometer (SCIEX, USA). Chromatographic separation was achieved using a BEH C18 column (2.1 mm × 10 cm, 1.7 μm; Waters). The QTRAP 6500+ mass spectrometer, equipped with an ESI Turbo Ion Spray interface, was operated with the following parameters: ion source temperature at 400°C, ion spray voltage (IS) at 4500 V (positive mode) and −4500 V (negative mode), and ion source gases I (GS1), II (GS2), and curtain gas (CUR) set to 60, 60, and 35 psi, respectively.

Quantitative analysis was performed using Skyline software (v.21.1.0.146). This software, configured for monoisotopic peaks with a mass tolerance of 0.6 Da and a mass range of 50–1500 Da, was used to generate a data matrix that included metabolite identification and quantification results for subsequent information analysis and processing. Quality control (QC) samples were generated from a mixture of sample extracts to assess the consistency of the sample under identical treatment. During data collection, a certain number of QC samples were inserted in test samples to monitor the repeatability of the analytical process. The results exported by Skyline software were imported into metaX^50^ for subsequent data preprocessing. For quality controls, we performed Probabilistic Quotient Normalization (PQN) to improve comparability between samples. QC-based robust LOESS signal correction (QC-RLSC) was then used to rectify experimental sample signal by local polynomial regression fitting, utilizing the QC sample as a reference. PCA analysis for all samples, including QC samples, was used to examine the overall distribution of samples in each group and the stability of the whole analysis process.

Metabolic pathways were identified using open database sources such as MetaboAnalyst, the Kyoto Encyclopedia of Genes and Genomes (KEGG) pathway database, and the Human Metabolome Database.

### 2.6. Animal experimental design

#### 2.6.1 Subjects and Drugs

2.6.1.

Four-week-old male C57BL/6J mice (initial weight 13–16 g; Gempharmatech Co., Ltd) were housed at a temperature of 22–23°C on a 12-h light/dark cycle with ad libitum access to water and food except during experimental sessions. The study was approved by the Biomedical Ethics Committee of Peking University. After the acclimatization period, mice were orally administered a cocktail of four antibiotics (ABX) for 7 days: ampicillin (200 mg/kg/day, Solarbio Cat#A6920), vancomycin (100 mg/kg/day, VIANEX S.A. PLANTC), neomycin (200 mg/kg/day, SIGMA Cat#N6386), and metronidazole (200 mg/kg/day, SIGMA Cat#M1547).^51,52^

Subsequently, mice were subjected to fecal microbiota transplantation (FMT) from ADHD patients with a low abundance of beneficial bacteria *Lactobacillus sanfranciscensi*s (group FMT-A), as *Lactobacillus sanfranciscensis* was the one we identified that had the most significant impact on ADHD symptoms. Mice administered fecal microbiota transplantation (FMT) from healthy donors served as the control group (group FMT-H). After three rounds of transplantation with a 5-day interval, the first time of behavioral experiments was conducted. Additionally, we randomly assigned them to three subgroups for rescue. The two experimental groups received interventions via drinking water throughout the entire experimental period to minimize dosage variability. Group FMT-A-R1 (*n* = 6) received *Lactobacillus sanfranciscensis* (~1 × 10^8^ organisms/mice/day), while group FMT-A-R2 (*n* = 6) received 150 mmol/L of sodium acetate (purity ≧99%, S8750, Sigma-Aldrich).^53,54^ The control group (FMT-A-C, *n* = 6) received an equivalent volume of PBS for 15 days, followed by a second round of behavioral assessments.

#### 2.6.2. Open field test

Behavioral experiments were conducted during the light period. Exploratory activity and hyperactivity behavior were measured using an Open-Field apparatus (40 × 40 × 25 cm). For the five days prior to the test, the mice were handled for 5 min every day, followed by a 5-min familiarization period in the box. Each mouse was placed in the center of the open-field apparatus. A square that was 10 cm from the wall was designated as the center zone. Traveled distance and time spent in the center zone of each animal were recorded for a single 4-min session with a video-imaging system.

#### 2.6.3. Five-choice serial reaction time task (5-CSRTT)

Mice were trained using a Five-Choice Serial Reaction Time Task (5-CSRTT) procedure according to a published protocol and previous studies.^55,56^ Sessions were conducted in standard chambers designed for the 5-CSRTT (Med Associates), housed within sound-attenuating, ventilated enclosures. Med-PC IV software (Med Associates) was used to set up a PC interface for controlling the session programs and collecting data.

During training, the LED of one of the five response holes will be on, and mice were trained to use their noses to locate the lit hole in order to receive a food pellet as a reward. Each session began with the illumination of the response holes light and the food magazine light, followed by the delivery of one food pellet (20 mg/pellet). Once the first food pellet was collected, the intertrial interval (ITI) commenced. At the end of the ITI, one of the five response holes on the chamber wall opposite the food magazine was illuminated for a brief period, known as the stimulus duration (SD). A correct response to this hole within the limited hold (LH) period resulted in the response holes light turning off, the food magazine light turning on, and delivery of one food pellet. After the pellet was collected, the next ITI began. The target stimulus varied pseudorandomly between trials to increase attentional load.

Training was divided into seven successive stages with specific criteria for ITI, SD, and LH, which had to be met for two consecutive days before progression to the next stage (Supplementary Fig. 6). A typical training session lasted 30 min or completed 100 trials, whichever came first. Responses into the non-target hole were considered incorrect responses, while responses during the ITI before target stimulus presentation were classified as premature responses, and a failure to respond was recorded as an omission. The average latency to correct responses and correct responses rate reflected the mice’s attention; the premature rate indicated the level of impulsivity.

#### 2.6.4. 16S rRNA gene analysis

Mice feces were collected before and after antibiotic treatment (ABX), after fecal microbiota transplantation (FMT), and after the rescue procedure (Figure 5A). At the time of collection, fecal samples were collected and immediately transported on dry ice within 20 min. Samples were stored in freezers at −80°C until analysis. After DNA extraction according to the protocols of Beijing Genomics Institute, the 16S rRNA gene was amplified using sequencing primers targeting the V3–V4 hypervariable region, and DNA was sequenced using the Illumina MiSeq platform. Microbiota bioinformatics were performed with QIIME 2 (https://qiime2.org/). Primer and adapter sequences were trimmed using cutadapt v2.6. Low-quality reads were filtered out using a sliding window approach (30 bp window, average quality <20). Reads shorter than 75% of their original length after trimming were discarded, as were reads containing ambiguous bases (N) or low-complexity sequences (≥10 consecutive identical bases). Amplicon Sequence Variants (ASVs) were then generated with 100% sequence similarity using the DADA2 method in QIIME2.

After obtaining genus-level ASVs, the data were normalized to ensure sample comparability; sequence count normalization was conducted through rarefaction to address uneven sequencing depths by the ‘rrarefy’ function in Vegan,^56^ with all samples being subsampled to the minimum sequence count. Specifically, we estimated the richness and evenness of the amplicon sequence variants by the number of observed features, Shannon, Simpson, and Chao1 index. We analyzed the similarities and differences in microbiota composition using Principal Coordinates Analysis (PCoA) based on Bray-Curtis distances. Additionally, we used PICRUSt to predict the functional profiles of the 16S rRNA gene sequences in the KEGG database. The representative ASV sequences were aligned against the Greengenes database. This allowed us to generate abundance tables for KEGG pathways.

### 2.7. Statistical analysis

Statistical analyses were performed using R software (version 4.2.0) and Statistical Product and Service Solutions (SPSS, version 26.0). Mean and standard deviation were utilized for continuous variables (including demographic data, dietary data, and psychometric parameters). Kruskal – Wallis tests and post-hoc Wilcoxon rank-sum pairwise tests, with p-values adjusted for multiple testing via the Benjamini – Hochberg method, were applied for inter-group comparisons. Correlation analysis was conducted using partial correlation analysis by the “spearman” method in the R package ppcor. When applicable, age, gender, BMI, SPM, site and principal component of diets were adjusted as covariates. A two-tailed p-value ≤0.05 was considered statistically significant.

#### 2.7.1. Taxonomic diversity analysis

We performed power analysis and created the species rarefaction curves by the function ‘specaccum’ in the R package vegan^57^ to predict species richness and determine whether the sample size was adequate. Data rarefaction was performed to allow for standardized inter-sample comparisons because sequencing depths varied among samples. All samples were subsampled to the minimum sequence count based on Taxonomy abundance matrix by the ‘rrarefy’ function in the R package vegan.^57^ The alpha diversity of the samples was estimated using the observed species, Shannon index, Simpson diversity, Pielou’s evenness index, and Phylogenetic diversity (PD) whole tree at the species level. Beta diversity between samples was estimated using the Bray – Curtis distance at the species level via the ‘vegdist’ function in the R package vegan.^57^ Anosim (Analysis of Similarities), MRPP (Multiple Response Permutation Procedure), and Adonis (Permutational Multivariate Analysis of Variance, PERMANOVA) based on the Bray-Curtis distance matrix were conducted using the ‘adonis,’ ‘MRPP,’ and ‘anosim’ functions from the R package vegan, with the permuted p-value obtained by 9,999 permutations. The Wilcoxon test with FDR correction was also used to compare alpha and beta diversity between groups.

To analyze the interrelationship between the microbiota present in stool samples and ADHD symptoms, we used redundancy analysis (RDA) from the R package vegan,^57^ using the abundance of normalized taxa and psychometric parameters (including ADHD-RS-IV, CBCL, and PSQ).

#### 2.7.2. Differential microbiota and metabolites analysis

To identify significant biomarkers, we compared all species between two groups using LEfSe analysis, which assisted in identifying genomic biomarkers that characterize statistical differences between biological groups.^58^ LEfSe employs an algorithm that performs the non-parametric Wilcoxon rank-sum test to identify bacterial taxa with significantly different relative abundances between groups. It applies linear discriminant analysis (LDA) to these identified taxa and further assesses the effect size of each. The significance thresholds were set at *p* < 0.05 and an LDA effect size (log10) >2. We also compared gene function of KEGG level 3 between groups using Wilcoxon rank-sum pairwise tests and Kruskal–Wallis H-test in STAMP,^59^ with p-values adjusted for multiple testing via the Benjamini – Hochberg method. Stamp plot was performed using OmicStudio tools at https://www.omicstudio.cn/tool.^60^

For metabolomics, after normalization to total peak intensity, the multivariate data were dimensionally reduced by principal component analysis (PCA) to analyze groupings, trends (intra-group and inter-group similarities and differences), and outliers in the data set. Using Partial Least Squares-Discriminant Analysis (PLS-DA) of the R package mixOmics^60^ and the classification performance of the PLS-DA model was evaluated through repeated 10-fold cross-validation. The variable importance in projection (VIP) values in the PLS-DA model were calculated to indicate each variable’s contribution to the classification. Through VIP, fold change, and Student’s t-test, we screened for differential metabolites. The q-values were obtained by adjusting the p-values of Student’s t-tests using the Benjamini–Hochberg method. Statistical significance and differentially expressed metabolites (DEMs) were defined as VIP ≥ 1, fold change ≥1.2 or ≤0.83 and q-value <0.05. Correlation analysis between symptoms and significant metabolites was conducted using the partial correlation method. For the identified intergroup differential metabolites, we also perform metabolic pathway enrichment analysis based on the KEGG database and construct the corresponding pathway enrichment network.

#### 2.7.3. Association analysis between metagenomics and metabolomics

To further investigate the correlation between gut microbiota function and fecal metabolites, we employed the MIMOSA model from the R package MIMOSA2.^61^ By proposing an indicator of microbial community metabolic capacity – CMP (community-wide metabolite potential) – a metabolic model was constructed to predict the impact of gut microbiota composition on metabolite concentrations. The detailed methods are as follows: (1) Map the taxonomic or gene abundance data to KEGG metabolic reference data to link the microbiome to metabolic reactions. (2) Calculate metabolic potential (MP) scores for each taxon, describing an approximate relative estimate of the effects of each taxon on each metabolite in each sample. The MP scores are a normalized linear sum of the abundances predicted to produce a metabolite minus those predicted to utilize it. (3) Total community-level metabolic potential (CMP) scores across all samples were compared. The rank-based regression model, which was recommended in MIMOSA2 as it can detect metabolite relationships more robustly and sensitively across a wider variety of data distributions, was used to assess whether CMPs are a significant predictor of metabolite levels, thereby linking species features with metabolite concentrations. The contributions of individual taxa to the model fit are calculated using a permutation-based approach, as are taxonomic contributors for metabolites with a model p-value less than 0.1. Metabolites with a significant positive association between CMP scores and metabolite levels are considered putatively microbiome-governed.

The causal mediation analysis was conducted to explore the influence and interactions of microbiota and metabolites, using the ‘mediate’ function in the R package mediation.^62^ One thousand resamples were generated using the bias-corrected percentile bootstrap method, and the mediating effect was deemed statistically significant if the 95% bootstrap CI did not contain 0. The test level was α = 0.05. Sensitivity analysis was also performed through the ‘medsens’ function to assess the sensitivity of analysis results to potential unmeasured confounding.

## 3. Results

### 3.1. Demographic and clinical feature

A total of 188 children and adolescents, consisting of 94 patients with ADHD (56 ADHD-I, 29 ADHD-C, 9 ADHD-HI) and 94 TDs, were recruited. Table 1 shows demographics, intelligence, and clinical symptoms of ADHD subgroup patients and typically developing controls. There were no significant differences among the four groups in age, height, weight, BMI (body mass index), gender, and Raven’s Standard Progress Matrice (SPM). As expected, the scores of inattention, hyperactivity, impulsivity, and total scores of ADHD-RS were higher in patients of the ADHD subgroup than in the group TD (Table 1). The normality and homogeneity of variance of the variables were tested, and the appropriate inter-group comparison methods were chosen (Supplementary Table 1). All participants completed a semi-quantitative food frequency questionnaire (FFQ) concerning their diet. The daily nutritional intake for each participant was calculated based on the frequency of food consumption and the nutritional content of each food item. After standardization, a principal component analysis (PCA) was performed. We selected the top dietary principal components (PC) with a minimum eigenvalue of 1.0 as covariates for further analysis (Supplementary Table 2).

**Table 1.** Demographics, intelligence, and clinical symptoms of ADHD subgroup and typically developing controls (TD) in the study cohort.

Characteristics	TD (*n* = 94)	IA (*n* = 56)	HA (*n* = 9)	C (*n* = 29)	Statistics	p-value
Age, years, mean ± S.D.	9.93 ± 0.20	9.77 ± 0.31	9.56 ± 0.60	9.45 ± 0.48	2.324	0.508
Height, m, mean ± S.D.	1.40 ± 0.01	1.44 ± 0.02	1.38 ± 0.03	1.42 ± 0.03	3.051	0.384
Weight, kg, mean ± S.D.	35.35 ± 1.07	39.13 ± 2.01	32.98 ± 1.91	40.39 ± 2.83	2.722	0.436
BMI, kg/m2, mean ± S.D.	17.86 ± 0.33	18.33 ± 0.61	17.22 ± 0.96	19.58 ± 1.01	2.898	0.408
Male, No. (%)	61 (64.9%)	33 (58.9%)	7 (77.8%)	21 (72.4%)	2.085	0.568
SPM, mean ± S.D.	109.16 ± 1.16	107.16 ± 1.42	103.63 ± 3.09	112.26 ± 2.19	4.246	0.236
**ADHD-RS-IV**						
Inattention	12.78 ± 0.42	25.98 ± 0.44	20.67 ± 0.97	27.66 ± 0.53	143.065	< 0.001^abc^
Hyperactivity	6.91 ± 0.20	10.63 ± 0.42	13.00 ± 0.37	15.41 ± 0.60	103.074	< 0.001^abce^
Impulsivity	4.98 ± 0.19	7.23 ± 0.32	11.00 ± 0.75	11.00 ± 0.29	100.779	< 0.001^abcde^
Total	24.67 ± 0.70	43.93 ± 0.73	44.67 ± 1.21	54.07 ± 1.01	145.982	< 0.001^abce^

BMI, body mass index; SPM, Raven’s Standard Progress Matrice; *p* value based on the Kruskal–Wallis test (continuous variables or the Wilcoxon rank-sum test for two groups) or Fisher’s exact test (categorical variables) for all groups. p^a^ < 0.05 for IA and TD; p^b^ < 0.05 for HA and TD; p^c^ < 0.05 for C and TD; p^d^ < 0.05 for IA and HA; p^e^ < 0.05 for IA and C.

### 3.2. ADHD symptoms are linked to changes in microbial community structure

We carried out shotgun metagenomic sequencing for all participants and obtained an average of 9,973,835,522 clean bases per sample. Detailed information on gut microbiota sequencing data was given in Supplementary Table 3. We employed species rarefaction curves to assess and predict the degree to which species richness in the community increases with sample size. The curve for groups TD, IA, and C exhibited a flat trajectory during the terminal phases, indicating that the sample sizes were adequate and the subsequent analyses were reasonable (Supplementary Fig. 1).

**Figure 1.**
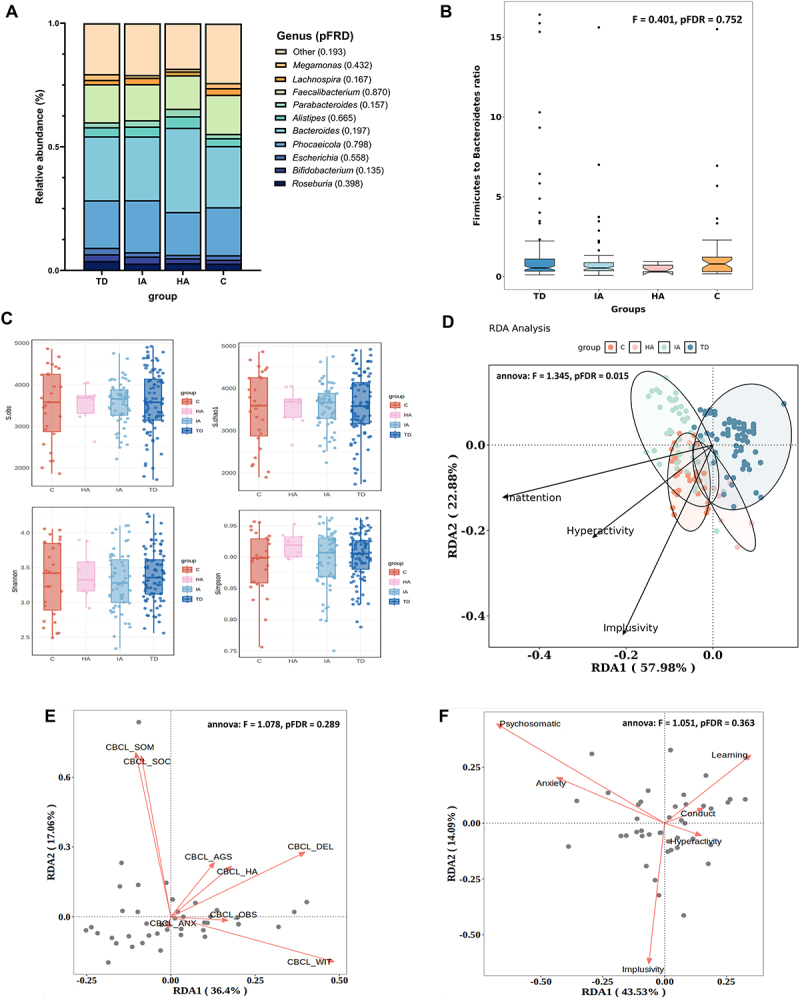
The overview of the microbiome profiles in three ADHD presentations and TD. (A) the top 10 taxa of relative abundance. The relative compositions of microbiota at the genus level were not significantly different among ADHD subgroups and group TD. The pFDR values of group comparisons are presented followed by legends of microbiota; (B) the Firmicutes to the bacteroidetes ratio. There was no group difference among ADHD subgroups and group TD using ANOVA corrected by FDR; (C) observed species, Shannon index, Simpson index, and Pielou’s evenness index for each group. Data are plotted as ±standard deviation; (D) RDA analysis of microbial community structure and ADHD symptoms in all participants; (E) RDA analysis of microbial community structure and CBCL. CBCL_SOM: somatic complaints of CBCL; CBCL_WIT: withdrawn of CBCL; CBCL_AGS: aggressive behavior of CBCL; CBCL_ANX: anxious/depressed symptoms; CBCL_SOC: social problems of CBCL; CBCL_HA: hyperactivity behaviors of CBCL; CBCL_DEL: delinquent behaviors of CBCL; (F) RDA analysis of microbial community structure and PSQ.

In terms of taxonomic diversity, we first compared the top 10 relative abundances of microbiota at the level of genus, the Firmicutes-to-Bacteroidetes ratio (F/B ratio), alpha diversity, and beta diversity between ADHD subgroups and TDs. The relative abundance of the top 10 genera of microbiota (Phocaeicola, Bacteroides, Alistipes, Parabacteroides, Faecalibacterium, Roseburia, Bifidobacterium, Escherichia, Lachnospira, Megamonas, and others) did not differ significantly (pFDR >0.05; Figure 1A). The F/B Ratio, which is widely acknowledged to have an important influence in maintaining normal intestinal homeostasis, was not differed significantly between groups (F = 0.401, *p* = 0.752; Figure 1B). We also found no significant differences in bacterial richness, evenness, and diversity of the microbial communities based on alpha-diversity indices (Figure 1C; Table 2). To assess the difference of microbial composition among groups, we also performed an analysis of similarities in the microbiome composition of beta diversity using various tests, including Anosim, MRPP, and Adonis (Table 3), yielding evidence indicating a significant microbial variability between groups (*R* = 0.0451, pFDR = 0.0676; A = 0.0053, pFDR = 0.0203; F = 1.6112, pFDR = 0.0257).

**Table 2.** Alpha diversity indexes for the ADHD subgroups and TDs.

Alpha diversity indexes	TD	IA	HA	C	F	Sig.
S.obs (observed species)	3579.76 ± 752.53	3526.29 ± 592.07	3559.44 ± 442.49	3455.31 ± 879.78	0.237	0.870
Shannon index	3.35 ± 0.41	3.28 ± 0.46	3.40 ± 0.33	3.30 ± 0.52	0.496	0.686
Simpson index	0.89 ± 0.05	0.89 ± 0.06	0.91 ± 0.02	0.88 ± 0.06	1.141	0.334
Pielou’s evenness index	0.41 ± 0.05	0.40 ± 0.05	0.42 ± 0.04	0.41 ± 0.06	0.551	0.648
PD whole tree	722.82 ± 165.11	710.34 ± 125.53	705.50 ± 92.81	700.12 ± 189.84	0.194	0.900

Total five indexes of alpha-diversity showed no group differences between ADHD subgroups and TD. S.obs (observed species) were used to estimate the indexes of the total number of species in the community. Shannon index, Simpson index, and Pielou’s evenness index were used to measure the microbial diversity in the sample. Phylogenetic diversity (PD) whole tree was the diversity index calculated the distance of the evolutionary tree.

**Table 3.** The significance tests of the community structure with greater between-group difference than the within-group difference.

Statistic methods	Values of level of between-group difference > within-group difference	Significance (pFDR value)
Anosim	*R* value: 0.04511	0.0676
MRPP	A score: 0.00529	0.0203
Adonis	F value: 1.61117	0.0257

Anosim similarity analysis is a non-parametric test to test whether the between-group difference is significantly greater than the difference within the group to judge whether the grouping is meaningful. MRPP analysis is similar to Anosim analysis, but the ordering method is different. It analyzes whether the microbial community structure difference between groups is significant. Adonis tests the differences between groups by partitioning the distance matrix among sources of variation and using a permutation test to assess the significance of these differences.

Furthermore, we used constrained ordination analysis to investigate the correlation between the microbial community structure and ADHD symptoms in all participants. To identify the optimal distribution of our data model (either a linear, unimodal, or bimodal distribution), we implemented a detrended correspondence analysis (DCA). The length value indicated that the linear model fitted our data, with the first axis (DCA1) having a length of 2.58 (less than 3) (Supplementary Table 4). We applied the redundancy analysis (RDA) to a linear model. We found that at the species level, Component 1 from the RDA explained 57.98% of the variance (22.88% with Component 2 scaling) (ANOVA like permutation test F = 1.345, pFDR = 0.015, Figure 1D). This indicated that ADHD symptoms, as environmental gradients, can explain changes in beta diversity of species, and the impact of three dimensions on microbial community structure is significant (inattention, $r^{2}$ = 0.2797, *p* < 0.001; hyperactivity, $r^{2}$ = 0.1732, *p* < 0.001; impulsivity, $r^{2}$ = 0.4011, *p* < 0.001; Table 4). For the participants with relevant information, we conducted analyses on the relationship between CBCL (Child Behavior Checklist), PSQ (Perceived Stress Questionnaire), and microbial community structure, respectively. The results indicated that neither the CBCL nor the PSQ was significantly related to the microbial community structure (ANOVA like permutation test F = 1.078, pFDR = 0.289, Figure 1E; ANOVA like permutation test F = 1.051, pFDR = 0.363, Figure 1F).

**Table 4.** The ANOVA like permutation test of the redundancy analysis (RDA) between the microbial community structure and ADHD symptoms.

Vectors	RDA1	RDA2	r2	p.adj
Inattention	−0.88047	−0.47409	0.2797	< 0.001
Hyperactivity	−0.60995	−0.79244	0.1732	< 0.001
Impulsivity	−0.32856	−0.94448	0.4011	< 0.001

RDA1 and RDA2 represent the proportion of species distribution explained by each axis in the analysis. The model’s R^2^ indicates the proportion of the total variance in the response variables that can be explained by the explanatory variables.

### 3.3. Specific taxa are linked to ADHD subtypes and core symptom domains

**Figure 2.**
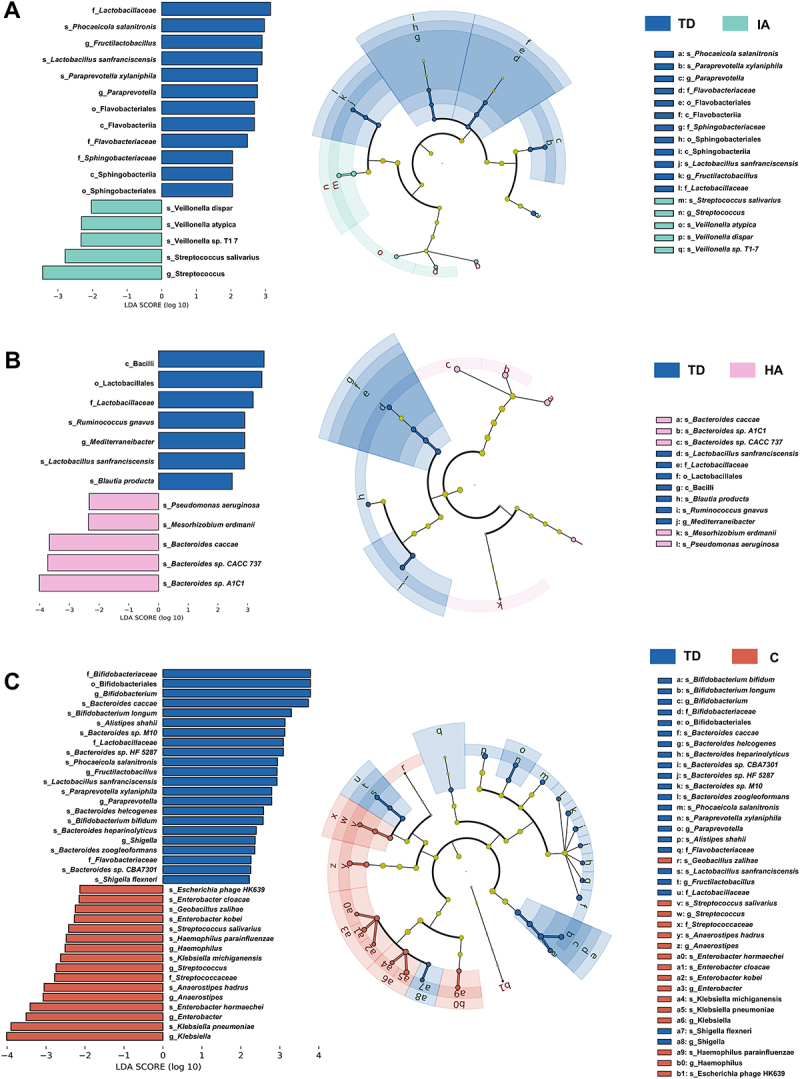
(A) a taxonomic cladogram and an LDA scores bar plot of identified differential taxa between group IA and TD; (B) a taxonomic cladogram and an LDA scores bar plot of identified differential taxa between group HA and TD; (C) a taxonomic cladogram and an LDA scores bar plot of identified differential taxa between group C and TD. The LDA score (log 10) > 2 and *p* < 0.05 are shown. The length of the bar indicates the effect size of each species. The circles from inside to outside represent different classification levels, and the size of each dot is proportional to its relative abundance. The colored taxa represent differential taxa between the ADHD subgroups and group TD. Blue, group TD-enriched; Green, group IA-enriched; Pink, group HA-enriched; red, group C-enriched.

We conducted a comprehensive analysis of the microbiota community variations from phylum to species levels between ADHD subgroups and group TD to identify potential evolutionary biomarkers linked to ADHD based on the gut microbiome. LEfSe analysis identified dominant bacterial biomarkers contributing to the group disparities. Specifically, 5 bacterial taxa were enriched in group IA and 12 were enriched in group TD when contrasting IA and TD (Figure 2A); 5 bacterial taxa were enriched in group HA, and 7 in group TD when contrasting HA and TD (Figure 2B); in addition, 16 bacterial taxa were enriched in group C and 22 in group TD when comparing C and TD (Figure 2C). These results suggest greater variation in the gut microbiota of patients in group C compared to those in group IA/HA.

Linear discriminant analysis of three ADHD subgroups and group TD concurrently revealed that family *Lactobacillaceae* and species *Lactobacillus sanfranciscensis* were enriched in TDs across all three comparative pairs. Additionally, in the comparison between Group C and Group TD, we identified that TDs exhibited a greater relative abundance in the microbiota from the order Bifidobacteriales to the evolutionary trees of *Bifidobacterium*-associated species, which have been demonstrated to have the capability to produce short-chain fatty acids in the human gut. In the two groups exhibiting prominent inattention symptoms (group IA and C), genus *Streptococcus* and species *Streptococcus salivarius* were identified as common harmful bacteria, while family *Flavobacteriaceae*, genus *Paraprevotella*, genus *Fructilactobacillus*, species *Paraprevotella xylaniphila*, and species *Phocaeicola salanitronis* were identified as common beneficial bacteria (Figure 2A, C). In the two groups with prominent hyperactivity-impulsivity symptoms (group HA and C), family *Lactobacillaceae* and species *Lactobacillus sanfranciscensis* were identified as common beneficial bacteria, while other microbiota showed few overlaps (Figure 2B, C).

**Figure 3.**
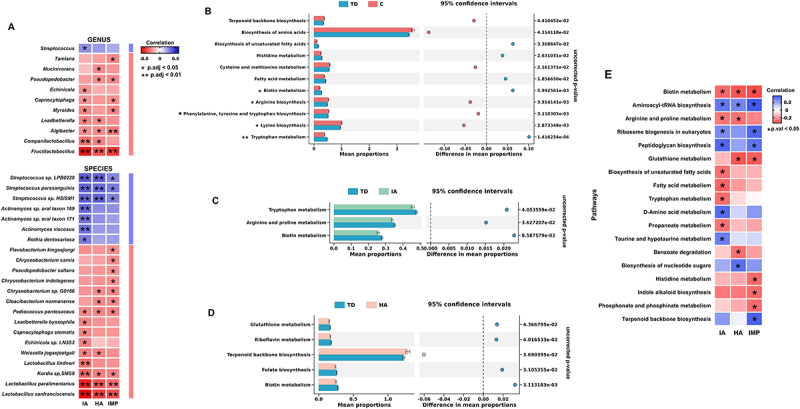
(A) differential correlation patterns of microbiota with ADHD symptoms. Pairs with pFDR < 0.05 are shown in the picture. The color of each grid represents the value of correlation coefficient. Blue color represents the negative correlation and red color represents the positive correlation; (B)-(D) the relative abundance of microbial function annotated by the KEGG database and significant microbial function among ADHD subgroups and group TD. The **symbol represents the pFDR < 0.05, while the *symbol represents the pFDR < 0.1; (E) differential correlation patterns of functional pathways with ADHD symptoms. Pairs with p-value < 0.05 are shown in the picture. The color of each grid represents the value of correlation coefficient. Blue color represents the negative correlation and red color represents the positive correlation.

To further elucidate the association between differential microbiota and symptoms, partial correlation analysis was carried out. After adjusting for various confounding factors, genus *Fructilactobacillus*, genus *Algibacter*, species *Lactobacillus sanfranciscensis*, species *Lactobacillus paralimentarius*, species *Kordia sp. SMS9*, and species *Pediococcus pentosaceus* showed a negative relationship with all three ADHD symptoms; species *Streptococcus sp. LPB0220*, species *Streptococcus parasanguinis*, and species *Streptococcus sp. HSISM1* showed a positive relationship with all three ADHD symptoms. Additionally, most of the identified species or genus were associated with only one or two specific ADHD symptoms, suggesting different microbial disruptions underlying each ADHD symptom domain (Figure 3A; Supplementary Table 5).

In the comparative analyses between ADHD subgroups and group TD, as well as the correlation analysis between ADHD symptoms and microbiota, the microbial features existing consistent results were considered more robust. Species *Lactobacillus sanfranciscensis* and family *Lactobacillaceae* exhibited significantly higher abundance in group TD across all three pairs of comparisons and were concurrently negatively correlated with all three ADHD symptoms in the correlation analysis. Furthermore, genus *Fructilactobacillus*, genus *Streptococcus*, and species *Streptococcus salivarius* showed differential abundance in group IA and group C when compared with group TD. Correspondingly, genus *Fructilactobacillus* was strongly correlated with all three ADHD symptoms, genus *Streptococcus* was correlated with inattention symptoms, and several species within the genus *Streptococcus* were correlated with the three ADHD symptoms.

### 3.4. ADHD microbial function alterations reveal different association with symptoms

After gene prediction, the total number of genes we obtained and the distribution of genes with different lengths were displayed in Supplementary Figure 2. First, we assessed the microbial function annotated by the KEGG database. We totally annotated the genes expressed by the microbiota to 5 pathways at Level 1, 29 pathways at Level 2, 202 pathways at Level 3, and 6935 KEGG orthology (Supplementary Fig. 3). We next focused on all metabolic pathways of Level 3 and employed STAMP to identify 11 differential pathways between group TD and group C (Figure 3B), 3 differential pathways between group TD and group IA (Figure 3C and 5) differential pathways between group TD and group HA (Figure 3D), most of which were related to amino acid metabolism and lipid metabolism.

In group C, tryptophan metabolism, biotin metabolism, histidine metabolism, fatty acid metabolism, and biosynthesis of unsaturated fatty acids were downregulated, while lysine biosynthesis, arginine biosynthesis, phenylalanine, tyrosine and tryptophan biosynthesis, and terpenoid backbone biosynthesis were upregulated (Figure 3B). In group IA, the expression of arginine and proline metabolism, biotin metabolism, and tryptophan metabolism were downregulated, with the latter two pathways showing consistent trends in group C (Figure 3C). In group HA, antioxidant-related pathways, including glutathione metabolism, riboflavin metabolism, folate biosynthesis, and biotin metabolism, were downregulated, while the expression of terpenoid backbone biosynthesis was upregulated (Figure 3D). These results suggest the potential impact of bacterial functions on the host.

Furthermore, we employed partial correlation analysis to investigate relationships between three domains of ADHD symptoms and KEGG pathways, adjusting for sex, age, BMI, SPM, site, and dietary variables. Results demonstrated that biotin metabolism showed negative correlations with all three symptoms, and aminoacyl-tRNA biosynthesis displayed positive correlations with all symptoms (Figure 3E; Supplementary Table 6). Most of the differential pathways exhibited correlations with only one or two specific symptoms, aligning with the prior correlation analysis between species and symptoms. For example, biosynthesis of unsaturated fatty acids, tryptophan metabolism, D-amino acid metabolism, propanoate metabolism, and taurine and hypotaurine metabolism were exclusively linked to inattention symptoms, while histidine metabolism, indole alkaloid biosynthesis, phosphonate and phosphinate metabolism, and terpenoid backbone biosynthesis were only linked to impulsivity symptoms.

Similarly, pathways demonstrating consistent results in both comparative analysis and correlation analysis were considered more robust. The biotin metabolism pathway showed increased abundance in group TD in all three pairs of comparisons, and correlation analysis also demonstrated that this pathway was negatively correlated with all three ADHD symptoms. The relative abundance of tryptophan metabolism was downregulated in group IA and group C and the pathway also showed a negative correlation with inattention symptoms. The relative abundance of terpenoid backbone biosynthesis was upregulated in group HA and group C, and the pathway also showed a positive correlation with impulsivity symptoms. In addition, group C, which exhibited symptoms of inattention, hyperactivity, and impulsivity, demonstrated differences in biosynthesis of unsaturated fatty acids, fatty acid metabolism, and histidine metabolism. Correspondingly, correlation analysis revealed that the first two pathways were associated with inattention, while histidine metabolism was associated with impulsivity.

### 3.5. ADHD metabolomic profiles reflect disturbances in fatty acid metabolism

To further elucidate the alterations in bacterial metabolism, we conducted untargeted metabolomics using fecal samples. The baseline comparison results between metabolomic subgroups and the full cohort are provided in Supplementary Table 7. No significant differences were observed in all these key variables, which supported the representativeness of the metabolomic subgroups. A total of 307 metabolites were identified, with classifications and the number of each class presented in Supplementary Figure 4. The comprehensive details of each identified metabolite are presented in Supplementary Table 8.

**Figure 4.**
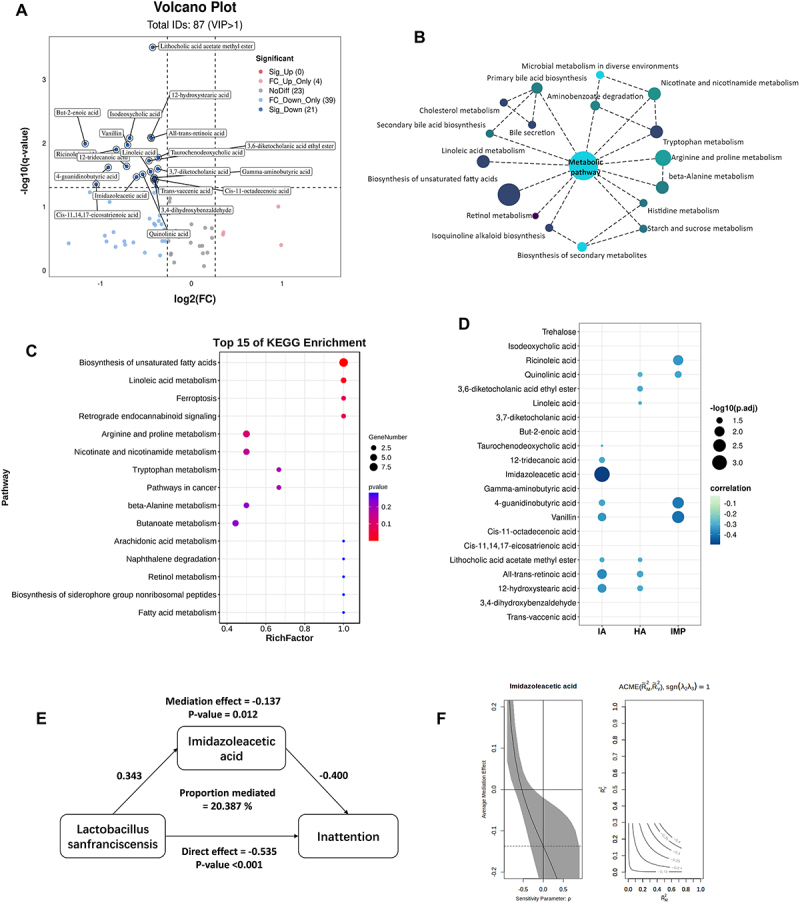
(A) Volcano plot of the different metabolites between group ADHD and group TD. The abscissa is the Fold change converted by log2, and the ordinate is the q-value converted by log10. The circles represent metabolites with a VIP > 1. Blue circles represent significant metabolites, while gray circles represent non-significant metabolites; (B) the KEGG pathway enrichment metabolic network of differential metabolites between groups, with node size representing the number of candidate genes in pathway; (C) bubble diagram for enrichment analysis based on the KEGG database. RichFactor is the ratio of differential metabolites annotated to the total number of identified metabolites annotated in a certain pathway; (D) differential correlation patterns of the metabolites with ADHD symptoms. The size of circles represents -log10(pFDR), pairs with pFDR < 0.05 are shown in the picture. The color of circle represents the value of correlation coefficient; (E) path diagram of the mediation analysis model of imidazoleacetic acid between *Lactobacillus sanfranciscensis* and inattention symptoms; (F) the sensitivity plots of causal mediation analysis. The average mediation effects are plotted as a function of the sensitivity parameter. Grey zones represent 95% confidence intervals of the estimated mediation effect across a range of hypothesized levels of residual confounding.

Based on the differential metabolite screening criteria, there were 21 downregulated metabolites identified in group ADHD compared to group TD (Figure 4A; Supplementary Table 9). Metabolic pathway enrichment analysis of differential metabolites was conducted based on the KEGG database. The 21 metabolites downregulated in the ADHD group were annotated, revealing significant decreases in the biosynthesis of unsaturated fatty acids and linoleic acid metabolism, both of which belong to the fatty acid metabolism pathway. Notably, several differential metabolites were also annotated to amino acid metabolism pathways, including arginine and proline metabolism, tryptophan metabolism, histidine metabolism, and beta-alanine metabolism (Figure 4B, C).

To explore the relationship between the significant differential metabolites and ADHD symptoms, we conducted a partial correlation analysis. After adjusting various confounding factors, we found that inattention symptom was negatively correlated with 12-hydroxystearic acid, all-trans-retinoic acid, lithocholic acid acetate methyl ester, vanillin, 4-guanidinobutyric acid, imidazoleacetic acid, 12-tridecanoic acid, and taurochendeoxycholic acid. Hyperactivity symptom was negatively correlated with 12-hydroxystearic acid, all-trans-retinoic acid, lithocholic acid acetate methyl ester, linoleic acid, 3,6-diketocholanic acid ethyl ester, and quinolinic acid. Impulsivity symptoms also had a negative correlation with vanillin, 4-guanidinobutyric acid, quinolinic acid, and ricinoleic acid (Figure 4D; Supplementary Table 10). Notably, imidazoleacetic acid demonstrated the strongest correlation with inattention symptoms (*r* = −0.493, p.adj < 0.001). These findings indicated the differences in metabolite profiles linked to various ADHD symptoms and suggested distinct underlying mechanisms contributing to the pathophysiology of ADHD symptom presentations.

### 3.6. Integrated analysis reveals SCFA deficiency and mediation effects in ADHD

To further explore the association between metagenomics and metabolomics between groups, we used the MIMOSA model to predict the impact of gut microbiota composition or function on metabolite concentrations. Based on the metabolic network approach, we then identified eight metabolites regulated by gut microbiome functions, which were annotated by KEGG orthology, including 4-Hydroxybenzoic acid, imidazoleacetic acid, hydroxyproline, 4-aminobutyric acid, 4-guanidinobutyric acid, L-asparagine, D-xylose, and pidolic acid. Importantly, imidazoleacetic acid, 4-aminobutyric acid, and 4-guanidinobutyric acid were previously identified as key differential metabolites between group ADHD and group TD, and all three are classified as short-chain fatty acids (SCFAs). It is noteworthy that the top producing pathways of these three metabolites are K00128, K00613, K01426, K00137, K01580, and K09473 (Table 5), corresponding to aldehyde dehydrogenase, glycine aminotransferase, amidase, aminobutyraldehyde dehydrogenase, glutamate decarboxylase, and gamma-glutamyl hydrolase, which are primarily involved in various amino acid and fatty acid processes. This result suggests that the gut microbiota may increase the risk of ADHD by regulating the expression of these aforementioned enzymes, leading to a decrease in the synthesis of the three short-chain fatty acids essential for maintaining gut homeostasis.

**Table 5.** Results of MIMOSA model to predict the impact of gut microbiota composition or function on metabolite concentrations.

ID	Name	R^2^	P-value	Top Producing Genes/Rxns	Top Utilizing Genes/Rxns
C00156	4-Hydroxybenzoic acid	0.142	0.0054	K00141 K01075 K01612 K03186	K03179 K06984
**C02835**	Imidazoleacetic acid	0.115	0.0130	K00128	
C01157	Hydroxyproline	0.096	0.0238	K00286 K00472	K12658 K23121
**C00334**	4-Aminobutyric acid	0.089	0.0299	K00128 K00137 K01580 K09473	
**C01035**	4-Guanidinobutyric acid	0.086	0.0332	K00613 K01426	
C01879	Pidolic acid	0.081	0.0383	K00682 K07232	K07160 K23123 K23124
C00152	L-Asparagine	0.077	0.0439	K01914	K01424 K01893 K22457
C00181	D-Xylose	0.071	0.0546	K01198	K00078 K14273

ID: KEGG compound ID for that metabolite; R^2^: Model R-squared; P-value: Model significance (drop-in-deviance test); Top Producing Taxa and Genes/Rxns: the genes or reactions with the largest contributors to variation in that metabolite, producing metabolite that contributed to the relevant CMP scores; Top Utilizing Taxa and Genes/Rxns: the genes or reactions a with the largest contributors to variation in that metabolite, utilizing that metabolite that contributed to the relevant CMP scores.

To investigate the influence and interactions of microbiota and metabolites on ADHD, we selected differential species and metabolites most strongly correlated with ADHD symptoms. The causal mediation analysis indicated a total effect of *Lactobacillus sanfranciscensis* on inattention symptoms of −0.672 (95% CI: −0.932 to −0.440), comprising a direct effect of −0.535 (95% CI: −0.757 to −0.315) and a mediation effect of −0.137 (95% CI: −0.269 to −0.048). Imidazoleacetic acid partially mediated these effects (p-value = 0.012), accounting for 20.387% of the total effect (Figure 4E). Sensitivity analyses indicated robustness against residual confounding of this result, with the rho at which ACME (average causal mediation effects) = 0 was −0.5 (Figure 4F). However, when hyperactivity and impulsivity were considered outcomes, the intervention–mediator interactions were not statistically significant (mediation effect = −0.068, p-value = 0.102; mediation effect = −0.100, p-value = 0.157; Supplementary Fig. 5; Supplementary Table 11). Overall, the results indicated that imidazoleacetic acid partially mediated the symptoms of inattention caused by a decrease in *Lactobacillus sanfranciscensis* but showed no significant effect for mediating symptoms of hyperactivity or impulsivity.

### 3.7. FMT confirms gut microbiota dysbiosis and metabolic dysfunction in mice

To verify the pathway from microbiota to metabolites and subsequently to ADHD symptoms, we conducted a fecal microbiota transplantation experiment in mice. Detailed information on the sequencing data of mice fecal samples was presented in Supplementary Table 12. The 16S rRNA analysis of mice fecal microbiota showed a significant reduction in alpha diversity following treatment with ABX. In terms of beta diversity, the microbiota of ABX-treated mice exhibited a distinct distribution pattern compared to wild-type mice (Supplementary Fig. 7). These findings validated the effectiveness of antibiotic treatment in altering the gut microbiota. After fecal microbiota transplantation from different sources, we observed that mice subjected to feces from ADHD patients with a low abundance of beneficial bacteria in group FMT-A exhibited hyperactive behavior (*t* = 2.098, *p* = 0.0476, *n* = 24; Supplementary Fig. 8A). In the Five-Choice Serial Reaction Time Task (5-CSRTT), mice in group FMT-A showed attention deficits, evidenced by an increase in average latency to correct response and a decrease in correct response rate (*t* = 2.245, *p* = 0.0351, *n* = 24; *t* = 3.209, *p* = 0.0040, *n* = 24; Supplementary Fig. 8B, C). However, no significant differences in impulsivity were observed when compared to group FMT-H (*t* = 0.1902, *p* = 0.8509, *n* = 24; Supplementary Fig. 8C).

**Figure 5.**
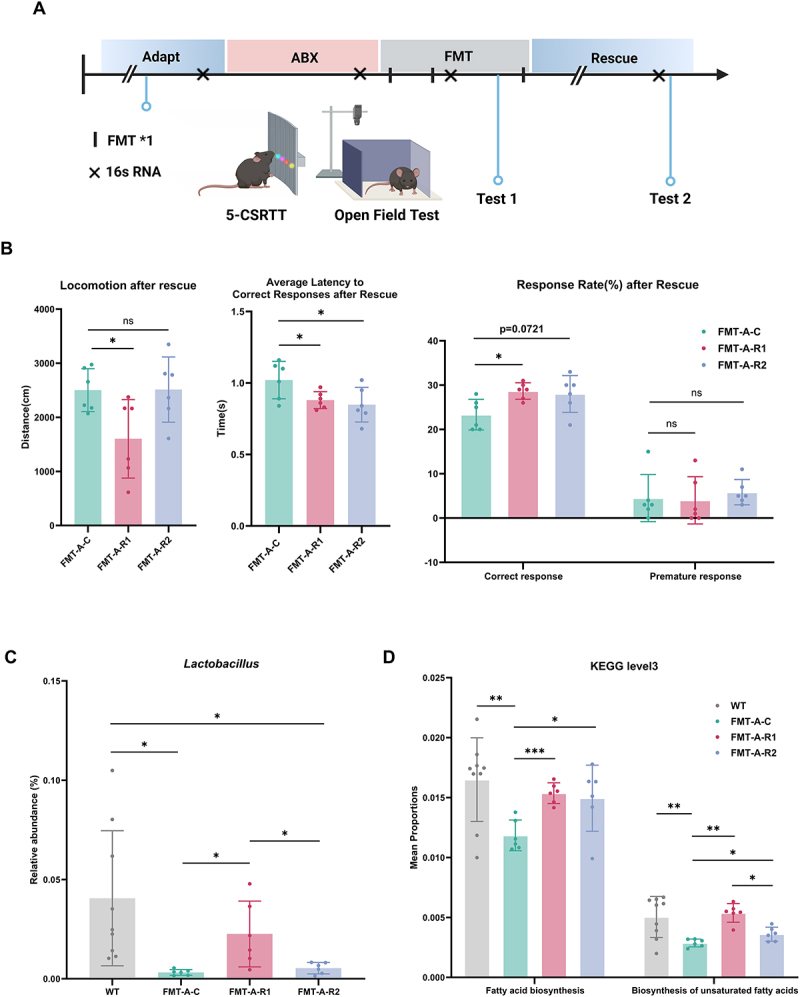
(A) workflow diagram of fecal microbiota transplantation (FMT) and rescue experiments in mice. ABX: orally administered a cocktail of four antibiotics, FMT: fecal microbiota transplantation; (B) performance of mice in the open field test and 5-choice serial reaction time task (5-CSRTT) following different rescue interventions; (C) relative abundance of the genus *Lactobacillus* in feces of mice after different rescue interventions; (D) mean proportions of predicted fatty acid synthesis-related KEGG pathways in feces of mice after different rescue interventions. The *symbol represents *p* < 0.05, the **symbol represents *p* < 0.01, the ***symbol represents *p* < 0.001.

In the rescue experiments for mice exhibiting ADHD-related symptoms after receiving FMT from ADHD patients, we found that compared to the control group (FMT-A-C), group FMT-A-R1, which received treatment with *Lactobacillus sanfranciscensis*, showed significant improvement in hyperactivity symptoms (*t* = 2.665, *p* = 0.0237, *n* = 12). In contrast, sodium acetate intervention failed to result in significant improvement in hyperactivity (*t* = 0.036, *p* = 0.9722, *n* = 12). Regarding attention, both *Lactobacillus sanfranciscensis* and sodium acetate alleviated symptoms (Figure 5B), evidenced by faster average correct response latency (*t* = 2.389, *p* = 0.0380, *n* = 12; *t* = 2.362, *p* = 0.0398, *n* = 12). In addition, the correct response rate was significantly elevated in group FMT-A-R1 (*t* = 3.145, *p* = 0.0104, *n* = 12), while group FMT-A-R2 showed a trend toward an increase in correct response rate (*t* = 2.011, *p* = 0.0721, *n* = 12). However, group FMT-A-R2 exhibited notable individual variations in response to treatment. Compared with FMT-A-C, neither group FMT-A-R1 nor FMT-A-R2 resulted in a statistically significant improvement in impulsive behavior (*t* = 0.1627, *p* = 0.8740, *n* = 12; *t* = 0.5408, *p* = 0.6005, *n* = 12). PCoA analysis based on Bray-Curtis distances revealed differences in microbial community structure between groups FMT-A-C, FMT-A-R1, and FMT-A-R2 (Supplementary Fig. 9).

Due to the limitations of 16S rRNA sequencing, we focused on the relative abundance of the genus *Lactobacillus*, which includes the target strain *Lactobacillus sanfranciscensis* (Figure 5C). Compared to group FMT-A-C, the relative abundance of *Lactobacillus* in group FMT-A-R1 significantly elevated (*t* = 2.847, *p* = 0.0174, *n* = 12), nearing levels similar to the group WT. In contrast, the FMT-A-R2 group showed no significant difference in *Lactobacillus* abundance compared to FMT-A-C (*t* = 1.611, *p* = 0.1383, *n* = 12). Furthermore, we compared the predicted KEGG pathways for fatty acid biosynthesis and biosynthesis of unsaturated fatty acids (Figure 5D; Supplementary Table 13). The results indicated that the mean proportions of both pathways were significantly higher in group FMT-A-R1 compared to group FMT-A-C (*t* = 5.575, *p* < 0.001, *n* = 12; *t* = 4.012, *p* = 0.002, *n* = 12), and there was no statistical difference when compared to group WT (*t* = 0.771, *p* = 0.454, *n* = 12; *t* = 0.519, *p* = 0.614, *n* = 12). In group FMT-A-R2, which was administered sodium acetate, the fatty acid biosynthesis pathway showed a notable increase compared to group FMT-A-C, which suggested the potential effectiveness of acetate intervention on fatty acid synthesis (*t* = 2.505, *p* = 0.031, *n* = 12), albeit with considerable individual variation. For the biosynthesis of unsaturated fatty acids pathway, the mean proportions in group FMT-A-R2 differed significantly from both group FMT-A-C and group FMT-A-R1 (*t* = 2.505, *p* = 0.031, *n* = 12; *t* = −4.091, *p* = 0.012, *n* = 12), indicating that acetate supplementation partially increased the biosynthesis of unsaturated fatty acids in the gut microbiota.

## 4. Discussion

Mental health includes mental, emotional, social, and behavioral functions, occurring along a continuum from optimal to poor.^63^ Among adolescents and children, even those typically developing who do not meet the diagnostic criteria for ADHD may exhibit traits associated with inattention and hyperactivity-impulsivity. Therefore, ADHD ought to be considered a disorder characterized by a continuous spectrum. Our study revealed that the differences between Group C and Group TD were more significant than those between Group IA and Group TD regarding both microbial species composition and gut microbiome functional expression, which could be attributed to the severity of symptoms. These results correspond with a previous study,^64^ which suggested that comparing patients with C-ADHD and typically developing controls may uncover more pronounced differences in bacterial diversity. Through a subgroup analysis of ADHD clinical symptoms, we obtained more comprehensive and thorough information.

The metagenomic data demonstrated gut dysbiosis in patients with ADHD. While comparing gut microbiota taxa between ADHD subgroups and group TD, there were no significant differences observed in alpha diversity between patients with ADHD and TDs, which aligns with several previous studies.^26–30^ Considering beta diversity, we used several methods to verify the differences of bray-curtis distance between groups. Our redundancy analysis further indicated that a portion of the variation in microbiota composition could be explained by the diagnostic groups and inattention, hyperactivity and impulsivity symptoms. However, redundancy analysis concerning the CBCL and PSQ did not reach statistical significance, probably due to the limited sample size of these two datasets.

To explore the relationship between taxa and ADHD symptoms, we employed LEfSe analysis between ADHD subgroups exhibiting different domains of symptoms and group TD, as well as correlation analysis between symptoms and microbiota. Microbial features that exhibited consistent results were deemed more robust. The LEfSe analysis of the ADHD subgroups and group TD revealed that the family *Lactobacillaceae* and the species *Lactobacillus sanfranciscensis* were enriched in group TD across three pairs of comparisons. When comparing with group C, we found that group TD exhibited a higher relative abundance from the order Bifidobacteriales to *Bifidobacterium-*associated species phylogenetic trees. Furthermore, we identified several bacteria that were strongly negatively correlated with ADHD symptoms in correlation analysis, such as *Lactobacillus sanfranciscensis, Lactobacillus paralimentarius, Kordia sp. SMS9, Lactobacillus lindneri* and *Weissella jogaejeotgal*. Notably, most of the probiotics identified through these two methods, including *Lactobacillus sanfranciscensis, Bifidobacterium longum, Bifidobacterium bifidum, Lactobacillus paralimentarius, Lactobacillus lindneri*, and *Weissella jogaejeotgali*, are all microorganisms capable of fermenting and producing short-chain fatty acids (SCFAs).^65^

Regarding the harmful bacteria enriched in ADHD groups or positively correlated with ADHD symptoms, both methods identified *Streptococcus*-associated species, such as *Streptococcus salivarius, Streptococcus sp. LPB0220, Streptococcus parasanguinis*, and *Streptococcus sp. HSISM*, which are recognized for colonizing the human oral cavity. When these bacteria manifest in other organs or locations, they can activate the host’s immune system, and their pathogenicity has also been reported.^66,67^ However, previous studies have reported high inconsistencies in bacterial taxa between ADHD patients and healthy controls. Results suggest that alterations in specific bacterial taxa observed in one cohort may not be replicated or may even exhibit contrary trends in another.^26–29,31–33^ These discrepancies may reflect differences in cohort size and participant characteristics, including age, sex, diet, medication usage, early life environment, maternal health, and other factors, as all these factors can affect the gut microbiota composition.

The analysis of predicted microbial functions revealed significant differences between ADHD subgroups and group TD. The most significant pathways include several amino acid-related pathways, such as tryptophan metabolism, phenylalanine, tyrosine and tryptophan biosynthesis, and lysine biosynthesis. As Li et al. reported, gut microbiota actively reshapes the host’s amino acid balance through the metabolism of intestinal amino acids.^68^ From this perspective, the dysregulation of amino acid metabolism observed in the gene expression of gut microbiota in our study further reflects an imbalance of amino acids within the host. Additionally, a widely accepted hypothesis regarding the pathophysiology of ADHD emphasizes the dysfunction of the monoamine neurotransmitters dopamine (DA), noradrenaline (NE), and serotonin (5-HT), which are involved in rewarding and motivational processes in the brain.^69–72^ Gut bacteria can affect the production of these neurotransmitters through the metabolism of amino acids such as tryptophan, phenylalanine, and tyrosine. And our study also observed a reduction in bacteria such as *Lactobacillus* and *Bifidobacterium*, which are related to GABA and dopamine,^69,73^ among ADHD patients. In metabolomics analysis, direct precursor amino acids of the monoamine neurotransmitters, such as L-phenylalanine, L-tyrosine, and L-tryptophan, showed no statistically significant intergroup differences, although tryptophan metabolism was identified in pathway enrichment analysis. These suggest that monoamine dysregulation may not represent a primary pathogenic driver in our cohort. Moreover, several antioxidant-related differential functional pathways were identified in the comparisons across groups, including biotin metabolism, glutathione metabolism, riboflavin metabolism, and folate biosynthesis, which may be associated with the pathogenesis of ADHD. The gut microbiota can enhance the host’s antioxidant capacity by producing antioxidant substances such as biotin and glutathione, which neutralize reactive oxygen free radicals and prevent cellular damage when the body is under oxidative stress. The terpenoid backbone biosynthesis pathway has been linked to impulsivity. Terpenoids constitute a broad and diverse family of secondary metabolites, and their roles in the gut microbiota–disease relationship remain unclear and require further research.

Our analysis of metagenomic functional pathways and fecal metabolites concurrently revealed a downregulation of both fatty acid metabolism and the biosynthesis of unsaturated fatty acids in ADHD. The most significantly enriched process was the biosynthesis of unsaturated fatty acids. Unsaturated fatty acids, including MUFAs, *n*–3 PUFAs, and *n*–6 PUFAs, are significant dietary nutrients potentially linked to neurodevelopment and brain function.^74–76^ Corresponding to our results, several studies have shown that individuals with ADHD have an abnormal essential fatty acid (EFA) status and significantly lower PUFA levels in blood.^77,78^ Stevens et al. also reported that diminished levels of total *n*-3 PUFAs in plasma phospholipids were associated with an increased incidence of behavioral problems, temper tantrums, and sleep disorders.^77^ In addition, polyunsaturated fatty acids (PUFAs) have been used in the treatment of ADHD and studies have confirmed that increasing PUFA intake leads to a significant improvement in ADHD symptoms.^78–82^ Overall, our findings suggest that the gut microbiota may play a role in the fatty acid metabolism dysfunction and unsaturated fatty acid deficiency underlying the abnormal EFA status in ADHD.

In addition, through the correlation analysis of metabolomics and metagenomics, we identified eight metabolites regulated by gut microbiota functions, three of which (imidazoleacetic acid, 4-aminobutyric acid, and 4-guanidinobutyric acid) also exhibited significant differences between groups. The three metabolites are categorized as short-chain fatty acids (SCFAs), and we have also identified several key enzymes that contribute significantly to their synthesis. This suggests that the impact of gut microbiota on ADHD pathogenesis may be mediated through the synthesis of SCFAs by regulating the expression of certain enzymes. Consistent with our findings, in previous studies, aberrant levels of SCFAs have been implicated in disease pathogenesis and are generally present in diminished quantities.^83^ SCFAs in the human body are generated through microbial fermentation of dietary fiber in the gut by beneficial bacteria, such as *Lactobacillus* and *Bifidobacterium*.^65^ These beneficial bacteria were enriched in TDs in our study and are known to possess neuroactive properties. For instance, SCFAs regulate immunity on the central nervous system and it has been recently demonstrated that the treatment of SCFAs could rescue impaired microglial function.^84^ Moreover, microbiota has also been shown to modulate neurotrophins involved in brain development and plasticity. Several researches clearly indicate that the gut microbiota can influence brain-derived neurotrophic factor (BDNF) levels mainly through the effect of SCFAs and this links the microbiota to neuroplasticity-related CNS systems.^85^ An increase in SCFAs through a fiber-rich diet was positively correlated with raising BDNF levels, which ultimately might improve ADHD symptoms.^70^ Our study further confirms the protective role of SCFAs in ADHD from the novel perspective of metagenomics and fecal metabolomics, which can serve as a valuable complement to the aforementioned studies.

In summary, in the analysis of linear discriminant analysis and symptom-related species from metagenomics, we identified probiotics primarily responsible for the production of short-chain fatty acids (SCFAs). This aligns with the findings from the association analysis between metagenomics and metabolomics, where the metabolites modulated by gut microbiota and significantly downregulated in cases were primarily SCFAs. Additionally, the important overlapped pathways in the metagenomic differential functional pathways and the differential metabolites enrichment analysis were fatty acid metabolism and biosynthesis of unsaturated fatty acids, with SCFAs being one of the key substances involved in these pathways. These findings collectively reveal a disturbance in fatty acid metabolism associated with the pathogenesis of ADHD. To further validate the hypothesis that SCFA-producing microbes affect ADHD pathogenesis through the mediation of SCFAs, we conducted a causal mediation analysis. The results confirmed that *Lactobacillus sanfranciscensis* has a partial mediating effect on inattention symptoms in ADHD through imidazoleacetic acid. Consequently, we undertook animal experiments of fecal microbiota transplantation to further validate our hypothesis. The results of our experiment in rodents suggest that for mice exhibiting ADHD-related symptoms after FMT of ADHD patients with a low abundance of probiotics, rescue with sodium acetate can alleviate attention deficit symptoms.

The main strengths of our study include a larger sample size of the Chinese population compared to previous studies. At the same time, we excluded participants with other psychiatric, neurological, gastrointestinal, and metabolic conditions, as well as those with active use of psychoactive drugs, antibiotics, and probiotics. Methodologically, we employed shotgun metagenomic sequencing, which offers greater species identification depth compared to 16S rRNA sequencing. In terms of analysis, we conducted subgroup analyses of ADHD clinical symptoms, and the comparison between group C and group TD provided more comprehensive and detailed information that may have been overlooked without the application of subgroup analysis. Regarding study content, previous research has mostly focused on measuring plasma concentrations of amino acids and fatty acids as well as analyzing their correlation with clinical symptoms. In contrast, our study, for the first time, investigates the pathogenic role of gut microbiota in ADHD through species variations, gene pathway expression and fecal-targeted metabolomics. Specifically, it reveals the involvement of gut microbiota in disrupted fatty acid and amino acid metabolism, particularly in the biosynthesis of unsaturated fatty acids. Furthermore, we formulated a hypothesis that gut microbiota affects ADHD symptoms through metabolic variations. We then subsequently conducted fecal microbiota transplantation experiment in mice to validate the hypothesis. This contributes to a deeper understanding of the mechanisms underlying metabolic disturbances in ADHD and addresses gaps in previous studies by linking the gut-brain axis mechanism to ADHD through gut microbiota profiles.

There are also some limitations to our study. First, excluding patients with gastrointestinal conditions or special diets may preclude participants with more aberrant microbiota compositions, thereby hindering the detection of correlations between gastrointestinal symptoms, food style, and microbial profiles. Second, due to the lower prevalence of the hyperactive/impulsive subtype in clinical settings, the sample size of group HA in our cohort might have been underpowered, and the sample size for each subgroup might be imbalanced. To address this problem, we emphasize the convergent evidence from our correlation analysis between microbial taxa, functions, and symptoms, which partially mitigates subgroup limitations. Third, we performed a cross-sectional study, which does not allow for confirming the causal directions between the correlational link between differential species, functional pathways, fecal metabolites, and the disease or symptoms. To address this problem, we conducted animal experiments; however, further longitudinal work in human populations should be undertaken to assess age-associated and other variations in gut microbiota.

Building upon our findings and the current progress of gut-brain axis research, large-scale prospective longitudinal studies are essential to track the dynamic development of the gut microbiome and its relationship with the onset, progression, and symptom fluctuation of ADHD. In parallel, well-designed randomized controlled trials evaluating the efficacy of dietary modifications, specific prebiotics, probiotics, or microbiota transplantation are warranted to explore the therapeutic potential of targeting microbial and metabolic dysbiosis in ADHD. Furthermore, mechanistic investigations into how gut microbes and their metabolites influence gut-brain signaling pathways, along with integrative multi-omics approaches encompassing host genetics, transcriptomics, and metabolomics, will be crucial for advancing our understanding of the complex biological underpinnings of ADHD and identifying novel therapeutic targets.

In conclusion, our study characterized the distinct gut microbiota and fetal metabolite profiles in patients with ADHD and its subtypes. We observed more gut microbial alterations in patients from group C compared to those in group IA or HA. Moreover, we employed metagenomics and fecal metabolomics techniques for the first time to elucidate the role of gut microbiota in the dysregulation of fatty acid and amino acid metabolism, with a particular focus on the biosynthesis of unsaturated fatty acids and SCFAs. Through causal mediation analysis of population sample data and mouse experiments of fecal microbiota transplantation, we further validated the pathway from microbiota to metabolites and subsequently to ADHD symptoms. The study provides new evidence supporting the association between the microbiota-gut-brain axis and ADHD and contributes to a deeper understanding of the mechanisms underlying these metabolic disturbances in ADHD.

## Supplementary Material

Supplemental Material

## Data Availability

The data that support the findings of this study are openly available in CNGB Sequence Archive (CNSA) of China National GeneBank DataBase (CNGBdb) at https://db.cngb.org/, reference number CNP0005363.
