# Supplementary material for: Symptom-specific gut microbial and metabolic profiles in ADHD reveal SCFA deficiency as a Key pathogenic mechanism
Source: Gut Microbes. 2025 Jul 27;17(1):2537755. doi: 10.1080/19490976.2025.2537755 (PMC12309550; doi:10.1080/19490976.2025.2537755)
Supplement: Supplemental Material [file KGMI_A_2537755_SM2147.docx]

**Supplementary Table 1** The normality test for the distribution of Demographics, intelligence, and ADHD symptoms through the Shapiro Wilk test

| Variables | group | Statistic | Sig. | group | Statistic | Sig. |
| --- | --- | --- | --- | --- | --- | --- |
| Inattention | IA | 0.974 | 0.256 | C | 0.897 | 0.008 |
|  | TD | 0.924 | <0.001 | HA | 0.964 | 0.835 |
| Hyperactivity | IA | 0.943 | 0.011 | C | 0.936 | 0.081 |
|  | TD | 0.893 | <0.001 | HA | 0.902 | 0.263 |
| Impulsivity | IA | 0.924 | 0.002 | C | 0.935 | 0.076 |
|  | TD | 0.769 | <0.001 | HA | 0.942 | 0.603 |
| total | IA | 0.970 | 0.171 | C | 0.932 | 0.062 |
|  | TD | 0.907 | <0.001 | HA | 0.966 | 0.858 |
| Age | IA | 0.907 | <0.001 | C | 0.913 | 0.020 |
|  | TD | 0.925 | <0.001 | HA | 0.927 | 0.452 |
| Height | IA | 0.970 | 0.183 | C | 0.901 | 0.010 |
|  | TD | 0.981 | 0.198 | HA | 0.963 | 0.827 |
| Weight | IA | 0.894 | <0.001 | C | 0.951 | 0.194 |
|  | TD | 0.935 | <0.001 | HA | 0.956 | 0.754 |
| BMI | IA | 0.894 | <0.001 | C | 0.901 | 0.010 |
|  | TD | 0.842 | <0.001 | HA | 0.902 | 0.265 |
| SPM | IA | 0.961 | 0.066 | C | 0.901 | 0.010 |
|  | TD | 0.972 | 0.039 | HA | 0.869 | 0.121 |

**Supplementary Table 2** The results of Principal Component Analysis (PCA) for dietary data in all participants

| Initial Eigenvalues | | | | | | | | Extraction Sums of Squared Loadings | | | |
| --- | --- | --- | --- | --- | --- | --- | --- | --- | --- | --- | --- |
| Component | | Total | % of Variance | | | Cumulative % | Total | | % of Variance | | Cumulative % |
| 1 | 16.755 | | | 57.775 | 57.775 | | 16.755 | | | 57.775 | 57.775 |
| 2 | 7.475 | | | 25.775 | 83.550 | | 7.475 | | | 25.775 | 83.550 |
| 3 | 1.802 | | | 6.213 | 89.763 | | 1.802 | | | 6.213 | 89.763 |
| 4 | 1.203 | | | 4.149 | 93.912 | | 1.203 | | | 4.149 | 93.912 |
| 5 | 0.934 | | | 3.221 | 97.133 | |  | | |  |  |
| 6 | 0.416 | | | 1.433 | 98.566 | |  | | |  |  |
| 7 | 0.355 | | | 1.226 | 99.792 | |  | | |  |  |
| 8 | 0.036 | | | 0.124 | 99.916 | |  | | |  |  |
| 9 | 0.020 | | | 0.069 | 99.985 | |  | | |  |  |
| 10 | 0.004 | | | 0.014 | 99.999 | |  | | |  |  |
| 11 | 0.000 | | | 0.001 | 100.000 | |  | | |  |  |

**Supplementary Table 3** Detailed information of the shotgun metagenomic sequencing data of fecal samples

| Sample | Raw  reads | Raw  bases | Clean reads | Clean  bases | Clean data rate | | q20 | q30 | gc | Reads remove host | Bases  remove  host | Host  rate |
| --- | --- | --- | --- | --- | --- | --- | --- | --- | --- | --- | --- | --- |
| Average | 77919937 | 11687990591 | 66651690 | 9997753648 | 87.66 | | 97.51 | 93.35 | 45.67 | 66492236 | 9973835522 | 0.239255 |
| A001 | 66660964 | 9999144600 | 66537574 | 9980636100 | 99.81 | | 97.60 | 94.27 | 48.51 | 66505568 | 9975835200 | 0.048102 |
| A002 | 66615704 | 9992355600 | 66471006 | 9970650900 | 99.78 | | 97.92 | 94.26 | 47.03 | 66446602 | 9966990300 | 0.036714 |
| A003 | 66884752 | 10032712800 | 66646832 | 9997024800 | 99.64 | | 97.41 | 93.79 | 45.56 | 66636662 | 9995499300 | 0.015260 |
| A004 | 66872650 | 10030897500 | 66582626 | 9987393900 | 99.57 | | 97.57 | 94.54 | 45.92 | 66510456 | 9976568400 | 0.108392 |
| A005 | 66731454 | 10009718100 | 66494354 | 9974153100 | 99.64 | | 98.53 | 95.43 | 49.30 | 66464286 | 9969642900 | 0.045219 |
| A006 | 66558814 | 9983822100 | 66452678 | 9967901700 | 99.84 | | 97.48 | 93.34 | 46.25 | 66438188 | 9965728200 | 0.021805 |
| A007 | 66778550 | 10016782500 | 66494486 | 9974172900 | 99.57 | 97.67 | | 94.12 | 45.68 | 66472672 | 9970900800 | 0.032806 |
| A008 | 66472472 | 9970870800 | 66370112 | 9955516800 | 99.85 | 97.73 | | 94.11 | 46.06 | 66355614 | 9953342100 | 0.021844 |
| A009 | 66658326 | 9998748900 | 66500530 | 9975079500 | 99.76 | 97.11 | | 93.55 | 44.30 | 66478828 | 9971824200 | 0.032634 |
| A010 | 66511278 | 9976691700 | 66407790 | 9961168500 | 99.84 | 97.77 | | 93.83 | 47.01 | 66393938 | 9959090700 | 0.020859 |
| A011 | 66562982 | 9984447300 | 66455032 | 9968254800 | 99.84 | 97.50 | | 93.79 | 45.04 | 66432790 | 9964918500 | 0.033469 |
| A012 | 66505972 | 9975895800 | 66375810 | 9956371500 | 99.80 | 97.45 | | 93.93 | 47.52 | 66361924 | 9954288600 | 0.020920 |
| A013 | 66479138 | 9971870700 | 66427002 | 9964050300 | 99.92 | 97.98 | | 93.70 | 45.48 | 66426306 | 9963945900 | 0.001048 |
| A014 | 66904166 | 10035624900 | 66648000 | 9997200000 | 99.62 | 97.39 | | 93.73 | 44.91 | 66634172 | 9995125800 | 0.020748 |
| A015 | 66694000 | 10004100000 | 66579646 | 9986946900 | 99.83 | 97.25 | | 93.46 | 46.22 | 66419316 | 9962897400 | 0.240809 |
| A016 | 66511530 | 9976729500 | 66405554 | 9960833100 | 99.84 | 97.11 | | 93.66 | 45.70 | 66163434 | 9924515100 | 0.364608 |
| A017 | 66518208 | 9977731200 | 66413882 | 9962082300 | 99.84 | 97.25 | | 93.54 | 46.26 | 66401272 | 9960190800 | 0.018987 |
| A018 | 67079166 | 10061874900 | 66766238 | 10014935700 | 99.53 | 97.36 | | 93.17 | 44.23 | 66744112 | 10011616800 | 0.033140 |
| A019 | 67026052 | 10053907800 | 66543644 | 9981546600 | 99.28 | 97.41 | | 93.17 | 44.2 | 66523108 | 9978466200 | 0.030861 |
| A020 | 66640588 | 9996088200 | 66539088 | 9980863200 | 99.85 | 97.42 | | 93.87 | 45.24 | 66511250 | 9976687500 | 0.041837 |
| A021 | 66723396 | 10008509400 | 66640680 | 9996102000 | 99.88 | 97.30 | | 93.35 | 46.8 | 66626056 | 9993908400 | 0.021945 |
| A022 | 66764266 | 10014639900 | 66512320 | 9976848000 | 99.62 | 97.83 | | 94.48 | 44.97 | 66496704 | 9974505600 | 0.023478 |
| A023 | 66546412 | 9981961800 | 66405690 | 9960853500 | 99.79 | 97.81 | | 94.31 | 46.56 | 66391222 | 9958683300 | 0.021787 |
| A024 | 66509632 | 9976444800 | 66338948 | 9950842200 | 99.74 | 97.32 | | 93.74 | 45.50 | 65439470 | 9815920500 | 1.355882 |
| A025 | 66571944 | 9985791600 | 66465902 | 9969885300 | 99.84 | 97.22 | | 92.94 | 48.56 | 66455088 | 9968263200 | 0.016270 |
| A026 | 66436738 | 9965510700 | 66325286 | 9948792900 | 99.83 | 97.26 | | 94.25 | 45.46 | 66309924 | 9946488600 | 0.023162 |
| A027 | 66701396 | 10005209400 | 66314392 | 9947158800 | 99.42 | 97.53 | | 93.95 | 44.17 | 66298254 | 9944738100 | 0.024336 |
| A028 | 66672696 | 10000904400 | 66540980 | 9981147000 | 99.80 | 97.39 | | 94.35 | 44.95 | 66493640 | 9974046000 | 0.071144 |
| A029 | 66592028 | 9988804200 | 66476386 | 9971457900 | 99.83 | 97.25 | | 93.11 | 45.26 | 66435860 | 9965379000 | 0.060963 |
| A030 | 66522610 | 9978391500 | 66426494 | 9963974100 | 99.86 | 97.72 | | 94.15 | 45.2 | 66403474 | 9960521100 | 0.034655 |
| A031 | 70800000 | 10620000000 | 66845978 | 10026896700 | 94.42 | 97.41 | | 93.08 | 44.96 | 66771092 | 10015663800 | 0.112028 |
| A032 | 66610568 | 9991585200 | 66471064 | 9970659600 | 99.79 | 97.64 | | 94.16 | 45.4 | 66333304 | 9949995600 | 0.207248 |
| A033 | 69920000 | 10488000000 | 66803994 | 10020599100 | 95.54 | 97.61 | | 93.57 | 44.97 | 66643928 | 9996589200 | 0.239605 |
| A034 | 69920000 | 10488000000 | 66960498 | 10044074700 | 95.77 | 97.58 | | 93.39 | 45.43 | 65792664 | 9868899600 | 1.744064 |
| A035 | 70560000 | 10584000000 | 66889240 | 10033386000 | 94.80 | 97.61 | | 93.64 | 45.23 | 66858030 | 10028704500 | 0.046659 |
| A036 | 82640000 | 12396000000 | 66879382 | 10031907300 | 80.93 | 97.97 | | 94.69 | 47.87 | 66808914 | 10021337100 | 0.105366 |
| A037 | 78880000 | 11832000000 | 66796742 | 10019511300 | 84.68 | 97.73 | | 94.09 | 48.64 | 66717636 | 10007645400 | 0.118428 |
| A038 | 71120000 | 10668000000 | 67001746 | 10050261900 | 94.21 | 97.44 | | 93.07 | 43.90 | 66984646 | 10047696900 | 0.025522 |
| A039 | 76160000 | 11424000000 | 67074044 | 10061106600 | 88.07 | 97.93 | | 94.44 | 48.11 | 66917600 | 10037640000 | 0.233241 |
| A040 | 81600000 | 12240000000 | 67103142 | 10065471300 | 82.23 | 98.12 | | 94.59 | 45.53 | 66813626 | 10022043900 | 0.431449 |
| A041 | 72720000 | 10908000000 | 67090404 | 10063560600 | 92.26 | 97.83 | | 94.06 | 46.87 | 67029154 | 10054373100 | 0.091295 |
| A042 | 94560000 | 14184000000 | 66878412 | 10031761800 | 70.73 | 98.29 | | 94.75 | 44.90 | 66804102 | 10020615300 | 0.111112 |
| A043 | 75360000 | 11304000000 | 66854908 | 10028236200 | 88.71 | 97.81 | | 94.03 | 45.15 | 66833872 | 10025080800 | 0.031465 |
| A044 | 73440000 | 11016000000 | 66938636 | 10040795400 | 91.15 | 97.77 | | 93.58 | 43.55 | 66866872 | 10030030800 | 0.107209 |
| A045 | 92000000 | 13800000000 | 66829226 | 10024383900 | 72.64 | 98.20 | | 94.81 | 47.57 | 66822358 | 10023353700 | 0.010277 |
| A046 | 81440000 | 12216000000 | 67008858 | 10051328700 | 82.28 | 98.29 | | 94.94 | 45.29 | 66982492 | 10047373800 | 0.039347 |
| A047 | 70160000 | 10524000000 | 66894278 | 10034141700 | 95.35 | 97.62 | | 91.63 | 43.47 | 66687104 | 10003065600 | 0.309704 |
| A048 | 74640000 | 11196000000 | 66940098 | 10041014700 | 89.68 | 98.12 | | 93.39 | 44.38 | 66576592 | 9986488800 | 0.543032 |
| A049 | 71680000 | 10752000000 | 66881972 | 10032295800 | 93.31 | 97.81 | | 92.67 | 45.21 | 66392122 | 9958818300 | 0.732410 |
| A050 | 72720000 | 10908000000 | 66904506 | 10035675900 | 92.00 | 97.92 | | 93.20 | 48.25 | 66896806 | 10034520900 | 0.011509 |
| A051 | 86720000 | 13008000000 | 66987660 | 10048149000 | 77.25 | 98.37 | | 94.45 | 47.95 | 66969512 | 10045426800 | 0.027092 |
| A052 | 71040000 | 10656000000 | 67084220 | 10062633000 | 94.43 | 97.91 | | 92.77 | 45.88 | 66834476 | 10025171400 | 0.372284 |
| A053 | 71120000 | 10668000000 | 66966674 | 10045001100 | 94.16 | 97.74 | | 92.34 | 45.32 | 66568558 | 9985283700 | 0.594499 |
| A054 | 73040000 | 10956000000 | 66885250 | 10032787500 | 91.57 | 97.85 | | 92.64 | 44.52 | 66875122 | 10031268300 | 0.015142 |
| A055 | 90000000 | 13500000000 | 66828102 | 10024215300 | 74.25 | 98.36 | | 94.37 | 43.79 | 66400466 | 9960069900 | 0.639904 |
| A056 | 80000000 | 12000000000 | 66821952 | 10023292800 | 83.53 | 97.94 | | 93.26 | 50.07 | 66805082 | 10020762300 | 0.025246 |
| A057 | 106160000 | 15924000000 | 66848276 | 10027241400 | 62.97 | 98.30 | | 94.70 | 49.28 | 66822850 | 10023427500 | 0.038035 |
| A058 | 92880000 | 13932000000 | 66924782 | 10038717300 | 72.06 | 98.41 | | 94.52 | 45.24 | 66916676 | 10037501400 | 0.012112 |
| A059 | 83040000 | 12456000000 | 66985074 | 10047761100 | 80.67 | 98.42 | | 94.73 | 47.31 | 66656898 | 9998534700 | 0.489924 |
| A060 | 72800000 | 10920000000 | 66969566 | 10045434900 | 91.99 | 97.86 | | 92.91 | 47.96 | 66965726 | 10044858900 | 0.005734 |
| A061 | 84560000 | 12684000000 | 66952132 | 10042819800 | 79.18 | 98.38 | | 94.12 | 45.78 | 66444178 | 9966626700 | 0.758682 |
| A062 | 75360000 | 11304000000 | 66997486 | 10049622900 | 88.90 | 97.88 | | 93.20 | 46.29 | 66995804 | 10049370600 | 0.002511 |
| A063 | 77200000 | 11580000000 | 66900810 | 10035121500 | 86.66 | 98.09 | | 93.56 | 47.92 | 66519828 | 9977974200 | 0.569473 |
| A064 | 80640000 | 12096000000 | 67022852 | 10053427800 | 83.11 | 98.11 | | 93.34 | 45.49 | 67005272 | 10050790800 | 0.026230 |
| A065 | 82480000 | 12372000000 | 66862648 | 10029397200 | 81.07 | 98.28 | | 94.05 | 43.62 | 66282248 | 9942337200 | 0.868048 |
| A066 | 89680000 | 13452000000 | 66788494 | 10018274100 | 74.47 | 98.14 | | 93.68 | 47.27 | 66659802 | 9998970300 | 0.192686 |
| A067 | 72960000 | 10944000000 | 67083364 | 10062504600 | 91.95 | 97.59 | | 92.42 | 46.61 | 67071090 | 10060663500 | 0.018297 |
| A068 | 78640000 | 11796000000 | 67081080 | 10062162000 | 85.30 | 97.89 | | 93.41 | 48.19 | 67072170 | 10060825500 | 0.013282 |
| A069 | 73454032 | 11018104800 | 64843078 | 9726461700 | 88.28 | 97.92 | | 93.59 | 48.73 | 64838970 | 9725845500 | 0.006335 |
| A070 | 78036890 | 11705533500 | 66047814 | 9907172100 | 84.64 | 98.06 | | 93.25 | 42.95 | 66002520 | 9900378000 | 0.068578 |
| A071 | 78160000 | 11724000000 | 66913528 | 10037029200 | 85.61 | 97.82 | | 92.80 | 43.98 | 66906798 | 10036019700 | 0.010058 |
| A072 | 73040000 | 10956000000 | 67015224 | 10052283600 | 91.75 | 97.82 | | 93.03 | 47.26 | 67004470 | 10050670500 | 0.016047 |
| A073 | 71520000 | 10728000000 | 66858016 | 10028702400 | 93.48 | 97.63 | | 92.84 | 47.04 | 66842820 | 10026423000 | 0.022729 |
| A074 | 71520000 | 10728000000 | 67030514 | 10054577100 | 93.72 | 97.50 | | 92.32 | 48.45 | 67013682 | 10052052300 | 0.025111 |
| A075 | 74240000 | 11136000000 | 67083062 | 10062459300 | 90.36 | 97.98 | | 93.50 | 47.18 | 67081418 | 10062212700 | 0.002451 |
| A076 | 73600000 | 11040000000 | 67068174 | 10060226100 | 91.13 | 97.73 | | 92.67 | 47.27 | 67065246 | 10059786900 | 0.004366 |
| A077 | 75200000 | 11280000000 | 66801234 | 10020185100 | 88.83 | 98.01 | | 93.31 | 45.93 | 66793780 | 10019067000 | 0.011158 |
| A078 | 88640000 | 13296000000 | 66880022 | 10032003300 | 75.45 | 98.18 | | 94.4 | 48.42 | 66854610 | 10028191500 | 0.037996 |
| A079 | 76000000 | 11400000000 | 67037624 | 10055643600 | 88.21 | 97.82 | | 92.82 | 46.3 | 66955568 | 10043335200 | 0.122403 |
| A080 | 77760000 | 11664000000 | 66903618 | 10035542700 | 86.04 | 98.02 | | 93.67 | 47.27 | 66885962 | 10032894300 | 0.026390 |
| A081 | 72960000 | 10944000000 | 67051342 | 10057701300 | 91.90 | 98.17 | | 93.59 | 46.90 | 67048316 | 10057247400 | 0.004513 |
| A082 | 72400000 | 10860000000 | 66910500 | 10036575000 | 92.42 | 98.19 | | 93.34 | 42.94 | 65796976 | 9869546400 | 1.664199 |
| A083 | 71440000 | 10716000000 | 67063592 | 10059538800 | 93.87 | 98.17 | | 93.33 | 42.94 | 66963178 | 10044476700 | 0.149730 |
| A084 | 74061822 | 11109273300 | 64068190 | 9610228500 | 86.51 | 98.30 | | 94.11 | 47.35 | 64050768 | 9607615200 | 0.027193 |
| A085 | 106080000 | 15912000000 | 66954370 | 10043155500 | 63.12 | 97.86 | | 94.14 | 45.74 | 66506504 | 9975975600 | 0.668912 |
| A086 | 76240000 | 11436000000 | 66982492 | 10047373800 | 87.86 | 98.35 | | 93.98 | 45.77 | 66755008 | 10013251200 | 0.339617 |
| A087 | 73120000 | 10968000000 | 66861406 | 10029210900 | 91.44 | 98.10 | | 93.27 | 44.58 | 66481636 | 9972245400 | 0.567996 |
| A088 | 73280000 | 10992000000 | 66808472 | 10021270800 | 91.17 | 98.19 | | 93.56 | 45.43 | 66783672 | 10017550800 | 0.037121 |
| A089 | 73015426 | 10952313900 | 66860054 | 10029008100 | 91.57 | 98.15 | | 93.32 | 45.51 | 66839110 | 10025866500 | 0.031325 |
| A090 | 110800000 | 16620000000 | 66898644 | 10034796600 | 60.38 | 98.71 | | 94.70 | 44.65 | 66747316 | 10012097400 | 0.226205 |
| A091 | 92000000 | 13800000000 | 66800430 | 10020064500 | 72.61 | 98.8 | | 95.22 | 43.87 | 66798998 | 10019849700 | 0.002144 |
| A092 | 88080000 | 13212000000 | 66937586 | 10040637900 | 76.00 | 98.34 | | 94.11 | 47.68 | 66932170 | 10039825500 | 0.008091 |
| A093 | 69440000 | 10416000000 | 67192168 | 10078825200 | 96.76 | 97.36 | | 92.69 | 44.48 | 67116372 | 10067455800 | 0.112805 |
| A094 | 95680000 | 14352000000 | 66961728 | 10044259200 | 69.99 | 98.54 | | 94.80 | 49.42 | 66598896 | 9989834400 | 0.541850 |
| H001 | 66678738 | 10001810700 | 66584288 | 9987643200 | 99.86 | 97.03 | | 92.85 | 46.08 | 66573898 | 9986084700 | 0.015604 |
| H002 | 66643200 | 9996480000 | 66509322 | 9976398300 | 99.80 | 97.54 | | 93.13 | 44.45 | 66136534 | 9920480100 | 0.560505 |
| H003 | 66533444 | 9980016600 | 66399972 | 9959995800 | 99.80 | 97.30 | | 93.72 | 45.68 | 66378774 | 9956816100 | 0.031925 |
| H004 | 66858576 | 10028786400 | 66655772 | 9998365800 | 99.70 | 97.47 | | 93.77 | 45.18 | 66627862 | 9994179300 | 0.041872 |
| H005 | 66723460 | 10008519000 | 66636470 | 9995470500 | 99.87 | 97.27 | | 93.66 | 46.35 | 66623112 | 9993466800 | 0.020046 |
| H006 | 66812390 | 10021858500 | 66620698 | 9993104700 | 99.71 | 97.25 | | 93.49 | 44.40 | 66613008 | 9991951200 | 0.011543 |
| H007 | 66840612 | 10026091800 | 66688828 | 10003324200 | 99.77 | 97.23 | | 93.08 | 46.16 | 66645908 | 9996886200 | 0.064359 |
| H008 | 66623980 | 9993597000 | 66522976 | 9978446400 | 99.85 | 97.28 | | 93.78 | 46.23 | 66504374 | 9975656100 | 0.027963 |
| H009 | 66446406 | 9966960900 | 66332190 | 9949828500 | 99.83 | 97.20 | | 93.69 | 44.23 | 66259046 | 9938856900 | 0.110269 |
| H010 | 66585740 | 9987861000 | 66504702 | 9975705300 | 99.88 | 97.12 | | 93.60 | 45.42 | 66475988 | 9971398200 | 0.043176 |
| H011 | 66457212 | 9968581800 | 66303670 | 9945550500 | 99.77 | 97.5 | | 94.01 | 43.79 | 66294120 | 9944118000 | 0.014403 |
| H012 | 66530758 | 9979613700 | 66441032 | 9966154800 | 99.87 | 97.39 | | 93.96 | 47.28 | 66418892 | 9962833800 | 0.033323 |
| H013 | 66565658 | 9984848700 | 66361844 | 9954276600 | 99.69 | 97.94 | | 94.62 | 43.15 | 55907518 | 8386127700 | 1.5753519 |
| H014 | 66796192 | 10019428800 | 66394184 | 9959127600 | 99.40 | 97.72 | | 94.17 | 45.25 | 66368368 | 9955255200 | 0.038883 |
| H015 | 66767262 | 10015089300 | 66508472 | 9976270800 | 99.61 | 98.42 | | 95.14 | 46.35 | 66481190 | 9972178500 | 0.041020 |
| H016 | 66721906 | 10008285900 | 66477362 | 9971604300 | 99.63 | 97.22 | | 93.52 | 44.19 | 66464224 | 9969633600 | 0.019763 |
| H017 | 66464586 | 9969687900 | 66321380 | 9948207000 | 99.78 | 97.22 | | 93.73 | 44.02 | 66297016 | 9944552400 | 0.036736 |
| H018 | 66472636 | 9970895400 | 66395014 | 9959252100 | 99.88 | 97.32 | | 93.87 | 48.23 | 66187860 | 9928179000 | 0.312002 |
| H019 | 66633232 | 9994984800 | 66517704 | 9977655600 | 99.83 | 97.11 | | 92.30 | 45.71 | 66494690 | 9974203500 | 0.034598 |
| H020 | 66484520 | 9972678000 | 66447372 | 9967105800 | 99.94 | 97.55 | | 92.73 | 45.85 | 65908424 | 9886263600 | 0.811090 |
| H021 | 66750904 | 10012635600 | 66317614 | 9947642100 | 99.35 | 97.63 | | 94.06 | 44.61 | 66304018 | 9945602700 | 0.020501 |
| H022 | 66647332 | 9997099800 | 66581056 | 9987158400 | 99.90 | 97.20 | | 93.61 | 47.38 | 66516740 | 9977511000 | 0.096598 |
| H023 | 66903278 | 10035491700 | 66589544 | 9988431600 | 99.53 | 97.97 | | 94.27 | 45.53 | 66587902 | 9988185300 | 0.002466 |
| H024 | 66449236 | 9967385400 | 66351382 | 9952707300 | 99.85 | 97.25 | | 93.84 | 46.13 | 66339782 | 9950967300 | 0.017483 |
| H025 | 66582042 | 9987306300 | 66425998 | 9963899700 | 99.77 | 97.27 | | 93.81 | 43.99 | 66412160 | 9961824000 | 0.020832 |
| H026 | 66572626 | 9985893900 | 66479736 | 9971960400 | 99.86 | 97.39 | | 93.77 | 47.35 | 66419434 | 9962915100 | 0.090707 |
| H027 | 66638622 | 9995793300 | 66564466 | 9984669900 | 99.89 | 97.34 | | 94.03 | 45.60 | 66535352 | 9980302800 | 0.043738 |
| H028 | 66585472 | 9987820800 | 66493716 | 9974057400 | 99.86 | 97.47 | | 93.32 | 44.76 | 66469272 | 9970390800 | 0.036761 |
| H029 | 66528998 | 9979349700 | 66437350 | 9965602500 | 99.86 | 98.03 | | 94.11 | 46.54 | 66436948 | 9965542200 | 0.000605 |
| H030 | 66848926 | 10027338900 | 66573500 | 9986025000 | 99.59 | 97.31 | | 93.63 | 43.55 | 66560962 | 9984144300 | 0.018833 |
| H031 | 76880000 | 11532000000 | 66892936 | 10033940400 | 87.01 | 98.20 | | 93.33 | 44.57 | 64303956 | 9645593400 | 3.870334 |
| H032 | 66947426 | 10042113900 | 66655430 | 9998314500 | 99.56 | 97.30 | | 93.69 | 44.98 | 66646800 | 9997020000 | 0.012947 |
| H033 | 157520000 | 23628000000 | 66811216 | 10021682400 | 42.41 | 98.81 | | 95.34 | 43.46 | 66775986 | 10016397900 | 0.052731 |
| H034 | 77120000 | 11568000000 | 66988432 | 10048264800 | 86.86 | 98.29 | | 93.68 | 45.20 | 66983828 | 10047574200 | 0.006873 |
| H035 | 89120000 | 13368000000 | 66846088 | 10026913200 | 75.01 | 98.47 | | 94.25 | 40.31 | 66544118 | 9981617700 | 0.451739 |
| H036 | 102320000 | 15348000000 | 66834666 | 10025199900 | 65.32 | 98.36 | | 93.74 | 42.91 | 66831954 | 10024793100 | 0.004058 |
| H037 | 103280000 | 15492000000 | 66912570 | 10036885500 | 64.79 | 98.26 | | 94.05 | 44.68 | 66910594 | 10036589100 | 0.002953 |
| H038 | 76320000 | 11448000000 | 67042052 | 10056307800 | 87.84 | 97.44 | | 92.25 | 46.11 | 66751232 | 10012684800 | 0.433787 |
| H039 | 84160000 | 12624000000 | 67154546 | 10073181900 | 79.79 | 97.78 | | 93.50 | 46.13 | 67138302 | 10070745300 | 0.024189 |
| H040 | 89600000 | 13440000000 | 67119256 | 10067888400 | 74.91 | 98.11 | | 94.35 | 44.47 | 67116762 | 10067514300 | 0.003716 |
| H041 | 109188956 | 16378343400 | 65732506 | 9859875900 | 60.20 | 98.45 | | 94.32 | 43.53 | 65727420 | 9859113000 | 0.007737 |
| H042 | 114160000 | 17124000000 | 66778126 | 10016718900 | 58.50 | 98.44 | | 94.51 | 46.16 | 66776786 | 10016517900 | 0.002007 |
| H043 | 81120000 | 12168000000 | 66842128 | 10026319200 | 82.40 | 98.06 | | 93.24 | 47.19 | 66651920 | 9997788000 | 0.284563 |
| H044 | 105520000 | 15828000000 | 66890150 | 10033522500 | 63.39 | 98.28 | | 93.83 | 42.82 | 66835056 | 10025258400 | 0.082365 |
| H045 | 121280000 | 18192000000 | 66922574 | 10038386100 | 55.18 | 98.47 | | 94.39 | 45.73 | 66921916 | 10038287400 | 0.000983 |
| H047 | 105440000 | 15816000000 | 66909604 | 10036440600 | 63.46 | 98.38 | | 94.35 | 48.19 | 66748216 | 10012232400 | 0.241203 |
| H048 | 100658268 | 15098740200 | 66639672 | 9995950800 | 66.20 | 98.37 | | 94.41 | 45.80 | 66625922 | 9993888300 | 0.020633 |
| H049 | 77200000 | 11580000000 | 66786144 | 10017921600 | 86.51 | 98.26 | | 93.61 | 44.40 | 66767622 | 10015143300 | 0.027733 |
| H050 | 86160000 | 12924000000 | 66822818 | 10023422700 | 77.56 | 98.42 | | 94.23 | 46.91 | 66774174 | 10016126100 | 0.072795 |
| H051 | 71040000 | 10656000000 | 66954318 | 10043147700 | 94.25 | 97.75 | | 92.37 | 45.08 | 66950644 | 10042596600 | 0.005487 |
| H052 | 85840000 | 12876000000 | 66989844 | 10048476600 | 78.04 | 98.33 | | 94.39 | 48.01 | 66923960 | 10038594000 | 0.098349 |
| H053 | 106640000 | 15996000000 | 66861580 | 10029237000 | 62.70 | 96.43 | | 91.95 | 41.36 | 66851748 | 10027762200 | 0.014705 |
| H054 | 75360000 | 11304000000 | 66905548 | 10035832200 | 88.78 | 95.87 | | 90.93 | 44.96 | 66890368 | 10033555200 | 0.022689 |
| H055 | 79963736 | 11994560400 | 64384004 | 9657600600 | 80.52 | 96.19 | | 91.41 | 37.80 | 64380280 | 9657042000 | 0.005784 |
| H056 | 96560000 | 14484000000 | 66829164 | 10024374600 | 69.21 | 96.45 | | 92.15 | 44.78 | 66827422 | 10024113300 | 0.002607 |
| H057 | 75040000 | 11256000000 | 67055548 | 10058332200 | 89.36 | 95.76 | | 89.76 | 47.28 | 66957160 | 10043574000 | 0.146726 |
| H058 | 117520000 | 17628000000 | 66959742 | 10043961300 | 56.98 | 96.90 | | 93.20 | 45.33 | 66954238 | 10043135700 | 0.008220 |
| H059 | 75377700 | 11306655000 | 65771520 | 9865728000 | 87.26 | 95.83 | | 91.08 | 45.25 | 65732886 | 9859932900 | 0.058740 |
| H060 | 77622518 | 11643377700 | 66208926 | 9931338900 | 85.30 | 95.71 | | 91.07 | 45.44 | 66196362 | 9929454300 | 0.018976 |
| H061 | 79525972 | 11928895800 | 64901290 | 9735193500 | 81.61 | 96.50 | | 92.03 | 45.37 | 64897408 | 9734611200 | 0.005981 |
| H062 | 81840000 | 12276000000 | 67018130 | 10052719500 | 81.89 | 96.16 | | 91.17 | 45.38 | 66942870 | 10041430500 | 0.112298 |
| H063 | 74720000 | 11208000000 | 66896592 | 10034488800 | 89.53 | 95.94 | | 91.17 | 46.52 | 66894338 | 10034150700 | 0.003369 |
| H064 | 77920000 | 11688000000 | 67069984 | 10060497600 | 86.08 | 96.06 | | 91.40 | 47.27 | 67067860 | 10060179000 | 0.003167 |
| H065 | 71685140 | 10752771000 | 61355712 | 9203356800 | 85.59 | 95.96 | | 90.80 | 43.94 | 61317536 | 9197630400 | 0.062221 |
| H066 | 110480000 | 16572000000 | 66836192 | 10025428800 | 60.50 | 96.61 | | 92.61 | 44.54 | 66828508 | 10024276200 | 0.011497 |
| H067 | 97680000 | 14652000000 | 66876296 | 10031444400 | 68.46 | 96.33 | | 92.23 | 47.18 | 66870772 | 10030615800 | 0.008260 |
| H068 | 114800000 | 17220000000 | 66867552 | 10030132800 | 58.25 | 96.78 | | 92.75 | 46.33 | 66866150 | 10029922500 | 0.002097 |
| H069 | 77040000 | 11556000000 | 66879882 | 10031982300 | 86.81 | 95.89 | | 91.05 | 45.34 | 66874276 | 10031141400 | 0.008382 |
| H070 | 83200000 | 12480000000 | 67099932 | 10064989800 | 80.65 | 96.29 | | 92.01 | 46.53 | 67089714 | 10063457100 | 0.015228 |
| H071 | 102160000 | 15324000000 | 67033848 | 10055077200 | 65.62 | 97.20 | | 93.35 | 42.53 | 67024682 | 10053702300 | 0.013674 |
| H072 | 91120000 | 13668000000 | 66985522 | 10047828300 | 73.51 | 96.09 | | 91.50 | 44.22 | 66983054 | 10047458100 | 0.003684 |
| H073 | 83040000 | 12456000000 | 66954344 | 10043151600 | 80.63 | 96.39 | | 91.75 | 44.95 | 66951874 | 10042781100 | 0.003689 |
| H074 | 87520000 | 13128000000 | 67232766 | 10084914900 | 76.82 | 96.56 | | 92.65 | 46.88 | 67225088 | 10083763200 | 0.011420 |
| H075 | 73680000 | 11052000000 | 67113354 | 10067003100 | 91.09 | 95.85 | | 91.07 | 44.8 | 67096058 | 10064408700 | 0.025771 |
| H076 | 70640000 | 10596000000 | 63979524 | 9596928600 | 90.57 | 95.66 | | 90.60 | 45.63 | 63906656 | 9585998400 | 0.113893 |
| H077 | 68343926 | 10251588900 | 62654452 | 9398167800 | 91.68 | 95.94 | | 91.24 | 44.47 | 62645760 | 9396864000 | 0.013873 |
| H078 | 89840000 | 13476000000 | 67005692 | 10050853800 | 74.58 | 96.36 | | 92.09 | 45.02 | 67003656 | 10050548400 | 0.003039 |
| H079 | 86480000 | 12972000000 | 67098052 | 10064707800 | 77.59 | 96.11 | | 92.27 | 50.44 | 67091146 | 10063671900 | 0.010292 |
| H080 | 78560000 | 11784000000 | 67043900 | 10056585000 | 85.34 | 96.24 | | 91.67 | 45.18 | 66884162 | 10032624300 | 0.238259 |
| H081 | 77840000 | 11676000000 | 66314292 | 9947143800 | 85.19 | 95.91 | | 91.16 | 43.30 | 66292770 | 9943915500 | 0.032455 |
| H082 | 86400000 | 12960000000 | 67120872 | 10068130800 | 77.69 | 96.5 | | 92.32 | 45.07 | 67046590 | 10056988500 | 0.110669 |
| H083 | 97760000 | 14664000000 | 66992592 | 10048888800 | 68.53 | 96.33 | | 92.22 | 45.73 | 66990600 | 10048590000 | 0.002973 |
| H084 | 77840000 | 11676000000 | 67101886 | 10065282900 | 86.20 | 96.23 | | 91.51 | 44.94 | 67099340 | 10064901000 | 0.003794 |
| H085 | 78720000 | 11808000000 | 67169622 | 10075443300 | 85.33 | 96.05 | | 91.52 | 45.65 | 67153422 | 10073013300 | 0.024118 |
| H086 | 85840000 | 12876000000 | 66875226 | 10031283900 | 77.91 | 96.05 | | 90.75 | 45.3 | 66825662 | 10023849300 | 0.074114 |
| H087 | 89760000 | 13464000000 | 67109698 | 10066454700 | 74.77 | 96.25 | | 92.28 | 49.37 | 67102638 | 10065395700 | 0.010520 |
| H088 | 86800000 | 13020000000 | 66959784 | 10043967600 | 77.14 | 96.53 | | 92.38 | 48.99 | 66888550 | 10033282500 | 0.106383 |
| H089 | 86960000 | 13044000000 | 66928946 | 10039341900 | 76.97 | 98.67 | | 94.76 | 43.63 | 66723838 | 10008575700 | 0.306456 |
| H090 | 73920000 | 11088000000 | 66999676 | 10049951400 | 90.64 | 97.64 | | 94.02 | 45.42 | 66996812 | 10049521800 | 0.004275 |
| H091 | 72480000 | 10872000000 | 66820602 | 10023090300 | 92.19 | 97.62 | | 94.08 | 44.83 | 66421604 | 9963240600 | 0.597118 |
| H092 | 71600000 | 10740000000 | 67089152 | 10063372800 | 93.70 | 97.57 | | 93.65 | 43.68 | 67073758 | 10061063700 | 0.022946 |
| H093 | 80080000 | 12012000000 | 66820858 | 10023128700 | 83.44 | 98.33 | | 93.90 | 44.79 | 66690758 | 10003613700 | 0.194700 |
| H094 | 76240000 | 11436000000 | 67018192 | 10052728800 | 87.90 | 98.19 | | 93.69 | 46.54 | 66381780 | 9957267000 | 0.949611 |

**Supplementary Table 4** Detrended correspondence analysis (DCA) before redundancy analysis (RDA)

|  | DCA1 | DCA2 | DCA3 | DCA4 |
| --- | --- | --- | --- | --- |
| Eigenvalues | 0.1895 | 0.1436 | 0.1211 | 0.09931 |
| Additive Eigenvalues | 0.1895 | 0.1439 | 0.11490 | 0.09851 |
| Decorana values | 0.1979 | 0.1429 | 0.1209 | 0.09345 |
| Axis lengths | 2.5798 | 1.9134 | 1.9224 | 1.89174 |

**Supplementary Table 5** The Correlation between differential microbiota and ADHD symptoms

| species | symptoms | estimate | statistic | p.value | p.adj |
| --- | --- | --- | --- | --- | --- |
| *Actinomyces sp oral taxon 169* | IA | 0.346 | 4.956 | 3.290E-06 | 2.016E-03 |
| *Actinomyces sp oral taxon 169* | HA | 0.270 | 3.776 | 2.156E-04 | 7.112E-02 |
| *Actinomyces sp oral taxon 169* | IMP | 0.261 | 3.643 | 2.182E-04 | 5.198E-02 |
| *Actinomyces sp oral taxon 171* | IA | 0.326 | 4.636 | 1.356E-05 | 5.814E-03 |
| *Actinomyces sp oral taxon 171* | HA | 0.256 | 3.560 | 4.737E-04 | 7.812E-02 |
| *Actinomyces sp oral taxon 171* | IMP | 0.223 | 3.083 | 1.471E-03 | 1.018E-01 |
| *Actinomyces viscosus* | IA | 0.335 | 4.790 | 6.908E-06 | 3.703E-03 |
| *Actinomyces viscosus* | HA | 0.255 | 3.549 | 4.924E-04 | 7.820E-02 |
| *Actinomyces viscosus* | IMP | 0.237 | 3.288 | 7.522E-04 | 7.331E-02 |
| *Capnocytophaga stomatis* | IA | -0.294 | -4.132 | 1.098E-04 | 3.362E-02 |
| *Capnocytophaga stomatis* | HA | -0.249 | -3.458 | 6.790E-04 | 9.099E-02 |
| *Capnocytophaga stomatis* | IMP | -0.247 | -3.425 | 4.709E-04 | 6.731E-02 |
| *Chryseobacterium carnis* | IA | -0.197 | -2.710 | 1.477E-02 | 3.423E-01 |
| *Chryseobacterium carnis* | HA | -0.219 | -3.019 | 2.901E-03 | 2.073E-01 |
| *Chryseobacterium carnis* | IMP | -0.279 | -3.906 | 8.208E-05 | 3.520E-02 |
| *Chryseobacterium indologenes* | IA | -0.242 | -3.363 | 1.884E-03 | 1.469E-01 |
| *Chryseobacterium indologenes* | HA | -0.259 | -3.602 | 4.079E-04 | 7.605E-02 |
| *Chryseobacterium indologenes* | IMP | -0.268 | -3.744 | 1.510E-04 | 4.980E-02 |
| *Chryseobacterium sp G0186* | IA | -0.258 | -3.598 | 8.282E-04 | 1.315E-01 |
| *Chryseobacterium sp G0186* | HA | -0.280 | -3.953 | 9.108E-05 | 3.906E-02 |
| *Chryseobacterium sp G0186* | IMP | -0.289 | -4.064 | 4.449E-05 | 2.726E-02 |
| *Cloacibacterium normanense* | IA | -0.232 | -3.207 | 3.167E-03 | 1.787E-01 |
| *Cloacibacterium normanense* | HA | -0.288 | -4.046 | 7.708E-05 | 3.673E-02 |
| *Cloacibacterium normanense* | IMP | -0.283 | -3.969 | 6.450E-05 | 3.073E-02 |
| *Echinicola sp LN3S3* | IA | -0.295 | -4.149 | 1.026E-04 | 3.384E-02 |
| *Echinicola sp LN3S3* | HA | -0.144 | -1.960 | 5.153E-02 | 4.193E-01 |
| *Echinicola sp LN3S3* | IMP | -0.188 | -2.568 | 6.834E-03 | 1.619E-01 |
| *Flavobacterium kingsejongi* | IA | -0.267 | -3.729 | 5.146E-04 | 9.193E-02 |
| *Flavobacterium kingsejongi* | HA | -0.251 | -3.496 | 5.950E-04 | 9.112E-02 |
| *Flavobacterium kingsejongi* | IMP | -0.271 | -3.795 | 1.249E-04 | 4.870E-02 |
| *Kordia sp SMS9* | IA | -0.360 | -5.198 | 1.083E-06 | 9.284E-04 |
| *Kordia sp SMS9* | HA | -0.308 | -4.353 | 2.240E-05 | 1.601E-02 |
| *Kordia sp SMS9* | IMP | -0.298 | -4.200 | 2.594E-05 | 2.780E-02 |
| *Lactobacillus lindneri* | IA | -0.335 | -4.778 | 7.294E-06 | 3.475E-03 |
| *Lactobacillus lindneri* | HA | -0.265 | -3.694 | 2.922E-04 | 6.961E-02 |
| *Lactobacillus lindneri* | IMP | -0.259 | -3.608 | 2.471E-04 | 5.297E-02 |
| *Lactobacillus paralimentarius* | IA | -0.516 | -8.104 | 1.537E-13 | 3.295E-10 |
| *Lactobacillus paralimentarius* | HA | -0.401 | -5.894 | 1.808E-08 | 3.876E-05 |
| *Lactobacillus paralimentarius* | IMP | -0.343 | -4.911 | 1.248E-06 | 2.675E-03 |
| *Lactobacillus sanfranciscensis* | IA | -0.577 | -9.509 | 2.413E-17 | 1.035E-13 |
| *Lactobacillus sanfranciscensis* | HA | -0.453 | -6.840 | 1.181E-10 | 5.065E-07 |
| *Lactobacillus sanfranciscensis* | IMP | -0.408 | -6.014 | 6.080E-09 | 2.607E-05 |
| *Leadbetterella byssophila* | IA | -0.293 | -4.128 | 1.115E-04 | 3.188E-02 |
| *Leadbetterella byssophila* | HA | -0.259 | -3.604 | 4.048E-04 | 7.890E-02 |
| *Leadbetterella byssophila* | IMP | -0.242 | -3.353 | 6.029E-04 | 6.987E-02 |
| *Pediococcus pentosaceus* | IA | -0.285 | -4.007 | 1.791E-04 | 4.801E-02 |
| *Pediococcus pentosaceus* | HA | -0.291 | -4.128 | 5.614E-05 | 3.009E-02 |
| *Pediococcus pentosaceus* | IMP | -0.298 | -4.193 | 2.670E-05 | 2.290E-02 |
| *Pseudopedobacter saltans* | IA | -0.234 | -3.245 | 2.802E-03 | 1.821E-01 |
| *Pseudopedobacter saltans* | HA | -0.250 | -3.478 | 6.326E-04 | 8.751E-02 |
| *Pseudopedobacter saltans* | IMP | -0.270 | -3.770 | 1.369E-04 | 4.893E-02 |
| *Rothia dentocariosa* | IA | 0.309 | 4.375 | 4.099E-05 | 1.598E-02 |
| *Rothia dentocariosa* | HA | 0.216 | 2.975 | 3.325E-03 | 2.067E-01 |
| *Rothia dentocariosa* | IMP | 0.227 | 3.143 | 1.213E-03 | 9.633E-02 |
| *Streptococcus parasanguinis* | IA | 0.369 | 5.337 | 5.616E-07 | 6.020E-04 |
| *Streptococcus parasanguinis* | HA | 0.350 | 5.022 | 1.217E-06 | 1.043E-03 |
| *Streptococcus parasanguinis* | IMP | 0.310 | 4.391 | 1.189E-05 | 1.700E-02 |
| *Streptococcus sp HSISM1* | IA | 0.357 | 5.150 | 1.354E-06 | 9.676E-04 |
| *Streptococcus sp HSISM1* | HA | 0.363 | 5.241 | 4.422E-07 | 4.740E-04 |
| *Streptococcus sp HSISM1* | IMP | 0.295 | 4.156 | 3.093E-05 | 2.211E-02 |
| *Streptococcus sp LPB0220* | IA | 0.376 | 5.459 | 3.121E-07 | 4.461E-04 |
| *Streptococcus sp LPB0220* | HA | 0.367 | 5.314 | 3.123E-07 | 4.464E-04 |
| *Streptococcus sp LPB0220* | IMP | 0.286 | 4.016 | 5.362E-05 | 2.874E-02 |
| *Weissella jogaejeotgali* | IA | -0.296 | -4.163 | 9.705E-05 | 3.468E-02 |
| *Weissella jogaejeotgali* | HA | -0.293 | -4.128 | 5.580E-05 | 3.418E-02 |
| *Weissella jogaejeotgali* | IMP | -0.265 | -3.705 | 1.743E-04 | 4.983E-02 |
|  |  |  |  |  |  |
| genus | symptoms | estimate | statistic | p.value | p.adj |
| *Algibacter* | IA | -0.268 | -3.746 | 8.412E-05 | 2.246E-02 |
| *Apibacter* | HA | -0.280 | -3.949 | 1.118E-04 | 3.733E-02 |
| *Apibacter* | IMP | -0.313 | -4.452 | 1.480E-05 | 9.720E-03 |
| *Capnocytophaga* | IA | -0.258 | -3.587 | 2.870E-04 | 4.789E-02 |
| *Capnocytophaga* | HA | -0.242 | -3.380 | 8.871E-04 | 9.110E-02 |
| *Capnocytophaga* | IMP | -0.271 | -3.801 | 1.961E-04 | 4.300E-02 |
| *Companilactobacillus* | IA | -0.340 | -4.871 | 2.418E-06 | 1.614E-03 |
| *Companilactobacillus* | HA | -0.300 | -4.258 | 3.290E-05 | 2.196E-02 |
| *Companilactobacillus* | IMP | -0.233 | -3.240 | 1.422E-03 | 6.920E-02 |
| *Echinicola* | IA | -0.260 | -3.617 | 1.386E-04 | 2.643E-02 |
| *Echinicola* | HA | -0.192 | -2.653 | 8.686E-03 | 1.657E-01 |
| *Echinicola* | IMP | -0.206 | -2.854 | 4.810E-03 | 1.320E-01 |
| *Fructilactobacillus* | IA | -0.552 | -8.904 | 5.592E-16 | 7.466E-13 |
| *Fructilactobacillus* | IMP | -0.405 | -5.989 | 1.090E-08 | 1.440E-05 |
| *Fructilactobacillus* | HA | -0.467 | -7.136 | 2.173E-11 | 2.901E-08 |
| *Leadbetterella* | IA | -0.293 | -4.128 | 5.575E-05 | 2.481E-02 |
| *Leadbetterella* | HA | -0.281 | -3.963 | 1.059E-04 | 4.710E-02 |
| *Leadbetterella* | IMP | -0.262 | -3.674 | 3.129E-04 | 5.140E-02 |
| *Mucinivorans* | IA | -0.156 | -2.129 | 3.460E-02 | 3.756E-01 |
| *Mucinivorans* | HA | -0.276 | -3.888 | 1.412E-04 | 3.770E-02 |
| *Mucinivorans* | IMP | -0.250 | -3.488 | 6.094E-04 | 5.340E-02 |
| *Myroides* | IA | -0.291 | -4.085 | 6.610E-05 | 2.206E-02 |
| *Myroides* | HA | -0.236 | -3.288 | 1.209E-03 | 9.491E-02 |
| *Myroides* | IMP | -0.287 | -4.056 | 7.370E-05 | 3.230E-02 |
| *Pseudopedobacter* | IA | -0.234 | -3.245 | 1.401E-03 | 8.908E-02 |
| *Pseudopedobacter* | HA | -0.270 | -3.789 | 2.054E-04 | 4.571E-02 |
| *Pseudopedobacter* | IMP | -0.285 | -4.017 | 8.590E-05 | 2.820E-02 |
| *Streptococcus* | IA | 0.260 | 3.733 | 8.777E-05 | 1.953E-02 |
| *Streptococcus* | HA | 0.236 | 3.289 | 1.206E-03 | 1.073E-01 |
| *Streptococcus* | IMP | 0.197 | 2.715 | 7.262E-03 | 1.540E-01 |
| *Tamlana* | IA | -0.187 | -2.568 | 1.104E-02 | 1.866E-01 |
| *Tamlana* | HA | -0.229 | -3.181 | 1.724E-03 | 1.001E-01 |
| *Tamlana* | IMP | -0.283 | -3.991 | 9.520E-05 | 2.500E-02 |

**Supplementary Table 6** The Correlation between KEGG pathways and ADHD symptoms

| Pathways | Symptom | estimate | p.value | statistic |
| --- | --- | --- | --- | --- |
| Biotin metabolism | IA | -0.294 | 0.028 | -2.258 |
| Biotin metabolism | HA | -0.327 | 0.014 | -2.545 |
| Biotin metabolism | IMP | -0.346 | 0.009 | -2.709 |
| Aminoacyl-tRNA biosynthesis | IA | 0.309 | 0.021 | 2.386 |
| Aminoacyl-tRNA biosynthesis | HA | 0.277 | 0.039 | 2.120 |
| Aminoacyl-tRNA biosynthesis | IMP | 0.380 | 0.004 | 3.020 |
| Arginine and proline metabolism | IA | -0.264 | 0.050 | -2.008 |
| Arginine and proline metabolism | HA | -0.277 | 0.039 | -2.115 |
| Arginine and proline metabolism | IMP | -0.235 | 0.081 | -1.777 |
| Ribosome biogenesis in eukaryotes | IA | 0.268 | 0.046 | 2.040 |
| Ribosome biogenesis in eukaryotes | HA | 0.239 | 0.076 | 1.810 |
| Ribosome biogenesis in eukaryotes | IMP | 0.349 | 0.008 | 2.737 |
| Peptidoglycan biosynthesis | IA | 0.348 | 0.009 | 2.724 |
| Peptidoglycan biosynthesis | HA | 0.213 | 0.115 | 1.601 |
| Peptidoglycan biosynthesis | IMP | 0.322 | 0.016 | 2.499 |
| Glutathione metabolism | IA | -0.132 | 0.332 | -0.979 |
| Glutathione metabolism | HA | -0.329 | 0.013 | -2.557 |
| Glutathione metabolism | IMP | -0.318 | 0.017 | -2.467 |
| Biosynthesis of unsaturated fatty acids | IA | -0.282 | 0.035 | 2.157 |
| Biosynthesis of unsaturated fatty acids | HA | -0.143 | 0.293 | -1.063 |
| Biosynthesis of unsaturated fatty acids | IMP | -0.153 | 0.259 | -1.140 |
| Fatty acid metabolism | IA | -0.302 | 0.024 | -2.331 |
| Fatty acid metabolism | HA | -0.195 | 0.150 | -1.461 |
| Fatty acid metabolism | IMP | -0.139 | 0.306 | -1.034 |
| Tryptophan metabolism | IA | -0.284 | 0.034 | -2.175 |
| Tryptophan metabolism | HA | -0.072 | 0.598 | -0.531 |
| Tryptophan metabolism | IMP | -0.018 | 0.896 | -0.131 |
| D-Amino acid metabolism | IA | 0.337 | 0.011 | 2.634 |
| D-Amino acid metabolism | HA | 0.097 | 0.475 | 0.720 |
| D-Amino acid metabolism | IMP | 0.234 | 0.083 | 1.768 |
| Propanoate metabolism | IA | -0.273 | 0.042 | -2.083 |
| Propanoate metabolism | HA | -0.129 | 0.342 | -0.958 |
| Propanoate metabolism | IMP | -0.174 | 0.199 | -1.300 |
| Taurine and hypotaurine metabolism | IA | 0.295 | 0.027 | 2.269 |
| Taurine and hypotaurine metabolism | HA | 0.070 | 0.607 | 0.518 |
| Taurine and hypotaurine metabolism | IMP | 0.090 | 0.508 | 0.666 |
| Benzoate degradation | IA | -0.100 | 0.464 | -0.737 |
| Benzoate degradation | HA | -0.280 | 0.036 | -2.146 |
| Benzoate degradation | IMP | -0.187 | 0.167 | -1.400 |
| Biosynthesis of nucleotide sugars | IA | 0.189 | 0.163 | 1.415 |
| Biosynthesis of nucleotide sugars | HA | 0.327 | 0.014 | 2.539 |
| Biosynthesis of nucleotide sugars | IMP | 0.240 | 0.075 | 1.813 |
| Histidine metabolism | IA | -0.145 | 0.286 | -1.077 |
| Histidine metabolism | HA | -0.166 | 0.222 | -1.235 |
| Histidine metabolism | IMP | -0.289 | 0.031 | -2.216 |
| Indole alkaloid biosynthesis | IA | -0.260 | 0.053 | -1.978 |
| Indole alkaloid biosynthesis | HA | -0.260 | 0.053 | -1.975 |
| Indole alkaloid biosynthesis | IMP | -0.290 | 0.030 | -2.223 |
| Phosphonate and phosphinate metabolism | HA | -0.092 | 0.498 | -0.682 |
| Phosphonate and phosphinate metabolism | IA | -0.164 | 0.228 | -1.218 |
| Phosphonate and phosphinate metabolism | IMP | -0.285 | 0.033 | -2.183 |
| Terpenoid backbone biosynthesis | IA | 0.232 | 0.085 | 1.752 |
| Terpenoid backbone biosynthesis | HA | 0.034 | 0.806 | 0.247 |
| Terpenoid backbone biosynthesis | IMP | 0.357 | 0.007 | 2.810 |

**Supplementary Table 7** The baseline comparison results between metabolomic subgroup and the full cohort

| Variable | ADHD Subgroup (n=31) | ADHD Full Cohort (n=94) | P-value (ADHD) | Control Subgroup (n=31) | Control Full Cohort (n=94) | P-value (Control) |
| --- | --- | --- | --- | --- | --- | --- |
| Age, years, mean ± S.D. | 10.19±0.41 | 9.65±0.24 | 0.193 | 10.45±0.36 | 9.93±0.20 | 0.216 |
| Height, m, mean ± S.D. | 1.43±0.03 | 1.43±0.02 | 0.893 | 1.41±0.02 | 1.40±0.01 | 0.184 |
| Weight, kg, mean ± S.D. | 38.97±2.56 | 38.93±1.50 | 0.957 | 37.20±1.84 | 35.35±1.07 | 0.313 |
| BMI, kg/m2, mean ± S.D. | 18.45±0.70 | 18.61±0.49 | 0.875 | 17.81±0.52 | 17.86±0.33 | 0.773 |
| Male, No. (%) | 21 (67.7%) | 61 (64.9%) | 0.772 | 20 (64.5%) | 61 (64.9%) | 0.970 |
| SPM, mean ± S.D. | 105.82±1.78 | 108.40±1.15 | 0.348 | 109.03±1.91 | 109.16±1.16 | 0.923 |
| **ADHD-RS-IV** |  |  |  |  |  |  |
| Inattention | 25.13±0.69 | 25.99±0.37 | 0.144 | 13.65±0.65 | 12.78±0.42 | 0.284 |
| Hyperactivity | 13.13±0.64 | 12.33±0.39 | 0.329 | 7.32±0.26 | 6.91±0.20 | 0.184 |
| Impulsivity | 9.35±0.48 | 8.76±0.29 | 0.364 | 5.13±0.26 | 4.98±0.19 | 0.510 |
| Total | 46.32±1.20 | 47.07±0.73 | 0.367 | 26.10±0.90 | 24.67±0.70 | 0.176 |

BMI, body mass index; SPM, Raven’s Standard Progress Matrice; For continuous variables, independent-samples t-tests were used for normally distributed data and Mann–Whitney U tests for non-normally distributed data. For categorical variables, Chi-square tests were applied.

**Supplementary Table 8** Detailed information of metabolites measured in high throughput targeted metabolome of fecal samples

| Metabolite ID | Name | Class |
| --- | --- | --- |
| HM4000001 | 12-dehydrocholic acid diacetate | Bile acids |
| HM4000002 | 12-ketolithocholic acid acetate, methyl ester | Bile acids |
| HM4000004 | 12-tridecanoic acid | Fatty acids |
| HM4000005 | 23-norcholic acid | Bile acids |
| HM4000006 | 23-norcholic acid diacetate | Bile acids |
| HM4000007 | 23-nordeoxycholic acid diacetate | Bile acids |
| HM4000008 | 23-nordeoxycholic acid diacetate, methyl ester | Bile acids |
| HM4000009 | 23-nordeoxycholic acid methyl ester | Bile acids |
| HM4000010 | 3,12-diketocholanic acid methyl ester | Bile acids |
| HM4000012 | 3,6-diketocholanic acid ethyl ester | Bile acids |
| HM4000013 | 3,6-diketocholanic acid methyl ester | Bile acids |
| HM4000014 | 3,7-diketocholanic acid | Bile acids |
| HM4000016 | 4-methylhexanoic acid | Organic acids |
| HM4000017 | 7-dehydrocholic acid methyl ester | Bile acids |
| HM4000018 | 7-ketolithocholic acid acetate methyl ester | Bile acids |
| HM4000021 | Alloisolithocholic acid | Bile acids |
| HM4000022 | Apocholic acid | Bile acids |
| HM4000023 | Beta-muricholic acid 3-acetate methyl ester | Bile acids |
| HM4000025 | Choleic acid | Bile acids |
| HM4000026 | Cholic acid methyl ester | Bile acids |
| HM4000027 | Dehydrocholic acid | Bile acids |
| HM4000028 | Dehydrolithocholic acid methyl ester | Bile acids |
| HM4000029 | Deoxycholic acid | Bile acids |
| HM4000030 | Deoxycholic acid methyl ester | Bile acids |
| HM4000031 | Dioxolithocholic acid | Bile acids |
| HM4000032 | Etiadienic acid | Bile acids |
| HM4000033 | Etiadienic acid 3-acetate | Bile acids |
| HM4000036 | Etianic acid | Bile acids |
| HM4000037 | Etienic acid | Bile acids |
| HM4000038 | Etienic acid acetate | Bile acids |
| HM4000040 | Glyco-lambda-muricholic acid | Bile acids |
| HM4000041 | Glycodehydrocholic acid | Bile acids |
| HM4000042 | Glycohyodeoxycholic acid | Bile acids |
| HM4000043 | Hyocholic acid methyl ester | Bile acids |
| HM4000044 | Hyodeoxycholic acid methyl ester | Bile acids |
| HM4000045 | Lithocholenic acid | Bile acids |
| HM4000046 | Lithocholenic acid acetate methyl ester | Bile acids |
| HM4000047 | Lithocholic acid acetate | Bile acids |
| HM4000048 | Lithocholic acid acetate methyl ester | Bile acids |
| HM4000049 | Nordeoxycholic acid | Bile acids |
| HM4000050 | Tauro-beta-muricholic acid | Bile acids |
| HM4000051 | Tauro-omega-muricholic acid | Bile acids |
| HM4000052 | Taurochenodeoxycholic acid | Bile acids |
| HM4000054 | Taurodeoxycholic acid | Bile acids |
| HM4000055 | Taurohyodeoxycholic acid | Bile acids |
| HM4000056 | Phosphoenolpyruvic acid | Organic acids |
| HM4000057 | 5-aminolevulinic acid | Amino acids |
| HMDB0000011 | 3-hydroxybutyric acid | Organic acids |
| HMDB0000019 | Alpha-ketoisovaleric acid | Organic acids |
| HMDB0000020 | P-hydroxyphenylacetic acid | Benzenoids |
| HMDB0000033 | Carnosine | Peptides |
| HMDB0000039 | Butyric acid | Fatty acids |
| HMDB0000043 | Betaine | Organic acids |
| HMDB0000045 | Adenosine monophosphate | Nucleosides |
| HMDB0000048 | Melibiose | Carbohydrates |
| HMDB0000060 | Acetoacetic acid | Organic acids |
| HMDB0000062 | Carnitine(c0) | Carnitines |
| HMDB0000064 | Creatine | Amino acids |
| HMDB0000072 | Cis-and trans-aconitic acid | Organic acids |
| HMDB0000092 | Dimethylglycine | Amino acids |
| HMDB0000094 | Citric acid | Organic acids |
| HMDB0000098 | D-xylose | Carbohydrates |
| HMDB0000112 | Gamma-aminobutyric acid | Amino acids |
| HMDB0000115 | Glycolic acid | Organic acids |
| HMDB0000118 | Homovanillic acid | Benzenoids |
| HMDB0000122 | D-glucose | Carbohydrates |
| HMDB0000123 | Glycine | Amino acids |
| HMDB0000124 | Fructose 6-phosphate | Carbohydrates |
| HMDB0000130 | Homogentisic acid | Benzenoids |
| HMDB0000134 | Fumaric acid | Organic acids |
| HMDB0000138 | Glycocholic acid | Bile acids |
| HMDB0000139 | Glyceric acid | Carbohydrates |
| HMDB0000150 | D-gluconolactone | Carbohydrates |
| HMDB0000152 | Gentisic acid | Benzenoids |
| HMDB0000156 | L-malic acid | Organic acids |
| HMDB0000159 | L-phenylalanine | Amino acids |
| HMDB0000161 | L-alanine | Amino acids |
| HMDB0000162 | L-proline | Amino acids |
| HMDB0000163 | D-maltose | Carbohydrates |
| HMDB0000167 | L-threonine | Amino acids |
| HMDB0000168 | L-asparagine | Amino acids |
| HMDB0000172 | L-isoleucine | Amino acids |
| HMDB0000176 | Maleic acid | Organic acids |
| HMDB0000177 | L-histidine | Amino acids |
| HMDB0000182 | L-lysine | Amino acids |
| HMDB0000187 | L-serine | Amino acids |
| HMDB0000190 | L-lactic acid | Organic acids |
| HMDB0000193 | Isocitric acid | Organic acids |
| HMDB0000194 | Anserine | Peptides |
| HMDB0000201 | L-acetylcarnitine | Carnitines |
| HMDB0000202 | Methylmalonic acid | Organic acids |
| HMDB0000208 | Oxoglutaric acid | Organic acids |
| HMDB0000214 | Ornithine | Amino acids |
| HMDB0000222 | Palmitoylcarnitine | Carnitines |
| HMDB0000223 | Oxalacetic acid | Organic acids |
| HMDB0000230 | N-acetylneuraminic acid | Carbohydrates |
| HMDB0000232 | Quinolinic acid | Organic acids |
| HMDB0000237 | Propanoic acid | Fatty acids |
| HMDB0000243 | Pyruvic acid | Organic acids |
| HMDB0000254 | Succinic acid | Organic acids |
| HMDB0000267 | Pyroglutamic acid | Amino acids |
| HMDB0000271 | Sarcosine | Amino acids |
| HMDB0000272 | Phosphoserine | Amino acids |
| HMDB0000273 | Thymidine | Nucleosides |
| HMDB0000291 | Vanillymandelic acid | Organic acids |
| HMDB0000328 | 12-ketolithocholic acid | Bile acids |
| HMDB0000391 | 7-dehydrocholic acid | Bile acids |
| HMDB0000407 | 2-hydroxy-3-methylbutyric acid | Fatty acids |
| HMDB0000415 | Beta-muricholic acid | Bile acids |
| HMDB0000434 | Homoveratric acid | Benzenoids |
| HMDB0000440 | 3-hydroxyphenylacetic acid | Benzenoids |
| HMDB0000448 | Adipic acid | Fatty acids |
| HMDB0000452 | L-alpha-aminobutyric acid | Organic acids |
| HMDB0000467 | 7-ketolithocholic acid | Bile acids |
| HMDB0000482 | Octanoic acid | Fatty acids |
| HMDB0000484 | Vanillic acid | Benzenoids |
| HMDB0000491 | 3-methyl-2-oxovaleric acid | Organic acids |
| HMDB0000500 | 4-hydroxybenzoic acid | Benzenoids |
| HMDB0000502 | 3-dehydrocholic acid | Bile acids |
| HMDB0000506 | Alpha-muricholic acid | Bile acids |
| HMDB0000509 | Senecioic acid | Organic acids |
| HMDB0000511 | Decanoic acid | Fatty acids |
| HMDB0000518 | Chenodeoxycholic acid | Bile acids |
| HMDB0000532 | Acetylglycine | Amino acids |
| HMDB0000535 | Caproic acid | Fatty acids |
| HMDB0000543 | Benzenebutanoic acid | Benzenoids |
| HMDB0000555 | 3-methyladipic acid | Fatty acids |
| HMDB0000557 | L-alloisoleucine | Amino acids |
| HMDB0000613 | Erythronic acid | Carbohydrates |
| HMDB0000617 | 2-furoic acid | Organic acids |
| HMDB0000619 | Cholic acid | Bile acids |
| HMDB0000622 | Ethylmalonic acid | Organic acids |
| HMDB0000623 | Dodecanedioic acid | Fatty acids |
| HMDB0000625 | Gluconic acid | Carbohydrates |
| HMDB0000631 | Glycodeoxycholic acid | Bile acids |
| HMDB0000634 | Citraconic acid | Fatty acids |
| HMDB0000637 | Glycochenodeoxycholic acid | Bile acids |
| HMDB0000638 | Dodecanoic acid | Fatty acids |
| HMDB0000641 | L-glutamine | Amino acids |
| HMDB0000651 | Decanoylcarnitine (c10) | Carnitines |
| HMDB0000661 | Glutaric acid | Organic acids |
| HMDB0000664 | Beta-hyodeoxycholic acid | Bile acids |
| HMDB0000666 | Heptanoic acid | Fatty acids |
| HMDB0000669 | Ortho-hydroxyphenylacetic acid | Benzenoids |
| HMDB0000673 | Linoleic acid | Fatty acids |
| HMDB0000678 | Isovalerylglycine | Amino acids |
| HMDB0000679 | L-homocitrulline | Amino acids |
| HMDB0000686 | Beta-ursodeoxycholic acid | Bile acids |
| HMDB0000687 | L-leucine | Amino acids |
| HMDB0000688 | Isovelarylcarnitine | Carnitines |
| HMDB0000689 | Isocaproic acid | Fatty acids |
| HMDB0000691 | Malonic acid | Organic acids |
| HMDB0000695 | Ketoleucine | Organic acids |
| HMDB0000696 | L-methionine | Amino acids |
| HMDB0000700 | Hydroxypropionic acid | Organic acids |
| HMDB0000703 | Mandelic acid | Benzenoids |
| HMDB0000705 | Hexanylcarnitine | Carnitines |
| HMDB0000707 | 4-hydroxyphenylpyruvic acid | Benzenoids |
| HMDB0000708 | Glycoursodeoxycholic acid | Bile acids |
| HMDB0000711 | Hydroxyoctanoic acid | Fatty acids |
| HMDB0000714 | Hippuric acid | Benzenoids |
| HMDB0000716 | L-pipecolic acid | Amino acids |
| HMDB0000719 | L-homoserine | Amino acids |
| HMDB0000720 | Levulinic acid | Organic acids |
| HMDB0000722 | Taurolithocholic acid | Bile acids |
| HMDB0000725 | 4-hydroxyproline | Amino acids |
| HMDB0000729 | Alpha-hydroxyisobutyric acid | Organic acids |
| HMDB0000733 | Hyodeoxycholic acid | Bile acids |
| HMDB0000742 | Homocysteine | Amino acids |
| HMDB0000746 | Hydroxyisocaproic acid | Fatty acids |
| HMDB0000748 | L-3-phenyllactic acid | Benzenoids |
| HMDB0000752 | Methylglutaric acid | Fatty acids |
| HMDB0000754 | 3-hydroxyisovaleric acid | Fatty acids |
| HMDB0000755 | Hydroxyphenyllactic acid | Phenylpropanoic acids |
| HMDB0000759 | Glycyl-l-leucine | Peptides |
| HMDB0000760 | Hyocholic acid | Bile acids |
| HMDB0000761 | Lithocholic acid | Bile acids |
| HMDB0000764 | Hydrocinnamic acid | Phenylpropanoic acids |
| HMDB0000766 | N-acetyl-l-alanine | Amino acids |
| HMDB0000779 | Phenyllactic acid | Benzenoids |
| HMDB0000783 | N-propionylglycine | Amino acids |
| HMDB0000784 | Azelaic acid | Fatty acids |
| HMDB0000792 | Sebacic acid | Fatty acids |
| HMDB0000806 | Myristic acid | Fatty acids |
| HMDB0000811 | Murocholic acid | Bile acids |
| HMDB0000812 | N-acetyl-l-aspartic acid | Amino acids |
| HMDB0000824 | Propionylcarnitine | Carnitines |
| HMDB0000840 | Salicyluric acid | Benzenoids |
| HMDB0000842 | Quinaldic acid | Carbohydrates |
| HMDB0000847 | Nonanoic acid | Fatty acids |
| HMDB0000848 | Stearylcarnitine(c18) | Carnitines |
| HMDB0000857 | Pimelic acid | Fatty acids |
| HMDB0000866 | N-acetyl-l-tyrosine | Amino acids |
| HMDB0000872 | Tetradecanedioic acid | Fatty acids |
| HMDB0000874 | Tauroursodeoxycholic acid | Bile acids |
| HMDB0000883 | L-valine | Amino acids |
| HMDB0000892 | Valeric acid | Fatty acids |
| HMDB0000893 | Suberic acid | Fatty acids |
| HMDB0000910 | Tridecanoic acid | Fatty acids |
| HMDB0000917 | Ursocholic acid | Bile acids |
| HMDB0000929 | L-tryptophan | Amino acids |
| HMDB0000930 | Trans-cinnamic acid | Benzenoids |
| HMDB0000933 | Traumatic acid | Fatty acids |
| HMDB0000943 | Threonic acid | Organic acids |
| HMDB0000946 | Ursodeoxycholic acid | Bile acids |
| HMDB0000947 | Undecanoic acid | Fatty acids |
| HMDB0000954 | Ferulic acid | Phenylpropanoic acids |
| HMDB0000956 | Tartaric acid | Carbohydrates |
| HMDB0000975 | Trehalose | Carbohydrates |
| HMDB0001043 | Arachidonic acid | Fatty acids |
| HMDB0001051 | Glyceraldehyde | Carbohydrates |
| HMDB0001336 | 3,4-dihydroxybenzeneacetic acid | Benzenoids |
| HMDB0001388 | Alpha-linolenic acid | Fatty acids |
| HMDB0001392 | P-aminobenzoic acid | Benzenoids |
| HMDB0001401 | Glucose 6-phosphate | Carbohydrates |
| HMDB0001488 | Nicotinic acid | Pyridines |
| HMDB0001491 | Pyridoxal 5'-phosphate | Benzenoids |
| HMDB0001514 | Glucosamine | Benzenoids |
| HMDB0001538 | 3-pyridylacetic acid | Pyridines |
| HMDB0001645 | L-norleucine | Amino acids |
| HMDB0001713 | M-coumaric acid | Benzenoids |
| HMDB0001844 | Methylsuccinic acid | Fatty acids |
| HMDB0001852 | All-trans-retinoic acid | Phenylpropanoic acids |
| HMDB0001856 | Protocatechuic acid | Benzenoids |
| HMDB0001867 | 4-aminohippuric acid | Benzenoids |
| HMDB0001870 | Benzoic acid | Benzenoids |
| HMDB0001877 | Valproic acid | Fatty acids |
| HMDB0001885 | 3-chlorotyrosine | Amino acids |
| HMDB0001904 | 3-nitrotyrosine | Amino acids |
| HMDB0001906 | 2-aminoisobutyric acid | Amino acids |
| HMDB0001955 | 3-phenylbutyric acid | Phenylpropanoic acids |
| HMDB0001987 | 2-hydroxy-2-methylbutyric acid | Amino acids |
| HMDB0001999 | Eicosapentaenoic acid epa | Fatty acids |
| HMDB0002000 | Myristoleic acid | Fatty acids |
| HMDB0002013 | Butyrylcarnitine | Carnitines |
| HMDB0002024 | Imidazoleacetic acid | Imidazoles |
| HMDB0002074 | 2,2-dimethylsuccinic acid | Fatty acids |
| HMDB0002085 | Syringic acid | Benzenoids |
| HMDB0002092 | Itaconic acid | Organic acids |
| HMDB0002096 | 3-indolebutyric acid | Indoles |
| HMDB0002107 | Phthalic acid | Benzenoids |
| HMDB0002108 | Methylcysteine | Amino acids |
| HMDB0002183 | Docosahexaenoic acid dha | Fatty acids |
| HMDB0002186 | (7z)-hexadecenoic acid | Fatty acids |
| HMDB0002210 | 2-phenylglycine | Amino acids |
| HMDB0002226 | Adrenic acid | Fatty acids |
| HMDB0002250 | Lauroylcarnitine | Carnitines |
| HMDB0002285 | 2-indolecarboxylic acid | Indoles |
| HMDB0002302 | 3-indolepropionic acid | Indoles |
| HMDB0002329 | Oxalic acid | Organic acids |
| HMDB0002372 | N-phenylacetylphenylalanine | Amino acids |
| HMDB0002393 | N-methyl-d-aspartic acid | Amino acids |
| HMDB0002511 | 3,4,5-trimethoxycinnamic acid | Phenylpropanoic acids |
| HMDB0002536 | Isodeoxycholic acid | Benzenoids |
| HMDB0002643 | Beta-(m-hydroxyphenyl)hydracrylic acid | Phenylpropanoic acids |
| HMDB0002925 | 8,11,14-eicosatrienoic acid | Fatty acids |
| HMDB0002931 | N-acetylserine | Amino acids |
| HMDB0003011 | O-acetylserine | Amino acids |
| HMDB0003070 | Shikimic acid | Organooxygen compounds |
| HMDB0003072 | Quinic acid | Organooxygen compounds |
| HMDB0003152 | N-methylnicotinamide | Pyridines |
| HMDB0003231 | Trans-vaccenic acid | Fatty acids |
| HMDB0003320 | Indole-3-carboxylic acid | Indoles |
| HMDB0003355 | 5-aminopentanoic acid | Amino acids |
| HMDB0003464 | 4-guanidinobutyric acid | Organic acids |
| HMDB0003681 | 4-acetamidobutanoic acid | Amino acids |
| HMDB0003966 | Selenomethionine | Amino acids |
| HMDB0004620 | N-a-acetyl-l-arginine | Amino acids |
| HMDB0005807 | Gallic acid | Benzenoids |
| HMDB0006029 | N-acetylglutamine | Amino acids |
| HMDB0006116 | 3-hydroxyhippuric | Benzenoids |
| HMDB0006331 | Cis,cis-muconic acid | Fatty acids |
| HMDB0006344 | Alpha-n-phenylacetyl-l-glutamine | Amino acids |
| HMDB0006528 | Docosapentaenoic acid dpa | Fatty acids |
| HMDB0010720 | But-2-enoic acid | Fatty acids |
| HMDB0011718 | 4-hydroxybenzaldehyde | Benzenoids |
| HMDB0011727 | Bicine | Organic acids |
| HMDB0011733 | Glycyl-glycine | Amino acids |
| HMDB0011745 | N-acetyl-l-methionine | Amino acids |
| HMDB0011756 | N-acetylleucine | Amino acids |
| HMDB0012308 | Vanillin | Benzenoids |
| HMDB0012328 | Palmitelaidic acid | Fatty acids |
| HMDB0013130 | Glutarylcarnitine(c5dc) | Carnitines |
| HMDB0013677 | 3,5-dihydroxybenzoic acid | Benzenoids |
| HMDB0013713 | N-acetyltryptophan | Amino acids |
| HMDB0029415 | S-carboxymethyl-l-cysteine | Amino acids |
| HMDB0029737 | Indole-3-carboxaldehyde | Indoles |
| HMDB0029738 | Indole-3-methyl acetate | Indoles |
| HMDB0031230 | 2-ethylhexanoic acid | Fatty acids |
| HMDB0031580 | 2-methylvaleric acid | Fatty acids |
| HMDB0032055 | N-acetyl-l-histidine | Amino acids |
| HMDB0032248 | 11-dodecenoic acid | Fatty acids |
| HMDB0032616 | Sinapic acid | Phenylpropanoic acids |
| HMDB0033161 | 2,6-pyridinedicarboxylic acid | Pyridines |
| HMDB0033724 | Undecylenic acid | Fatty acids |
| HMDB0034297 | Ricinoleic acid | Fatty acids |
| HMDB0037115 | 2-hydroxy-4-(methylthio) butanoate | Fatty acids |
| HMDB0038340 | N-(p-coumaroyl) serotonin | Indoles |
| HMDB0059965 | 3,4-dihydroxybenzaldehyde | Benzenoids |
| HMDB0060039 | Cis-11,14,17-eicosatrienoic acid | Fatty acids |
| HMDB0060460 | Cis-4-hydroxy-d-proline | Amino acids |
| HMDB0061706 | 12-hydroxystearic acid | Fatty acids |
| HMDB0061714 | 13c,16c-docosadienoic acid | Fatty acids |
| HMDB0062248 | Myristelaidic acid | Fatty acids |
| HMDB0062477 | 2-deoxyglucose | Carbohydrates |
| HMDB0094701 | N-acetylproline | Amino acids |
| HMDB0240219 | Cis-11-octadecenoic acid | Fatty acids |

**Supplementary Table 9** The variable importance in projection (VIP) values in the PLS-DA model

| Name | KEGG ID | Fold change | q-value | VIP |
| --- | --- | --- | --- | --- |
| Cis-11,14,17-eicosatrienoic acid | C16522 | 0.4834 | 0.044326 | 2.887948 |
| Trehalose | C01083 | 0.7638 | 0.048019 | 2.628624 |
| But-2-enoic acid | C01771 | 0.4447 | 0.010219 | 2.449514 |
| Linoleic acid | C01595 | 0.7326 | 0.028130 | 2.408376 |
| 3,6-diketocholanic acid ethyl ester | NA | 0.7237 | 0.018876 | 2.369254 |
| All-trans-retinoic acid | C00777 | 0.7365 | 0.008377 | 2.207679 |
| 12-hydroxystearic acid | C03042 | 0.7337 | 0.008154 | 2.176993 |
| Imidazoleacetic acid | C02835 | 0.658 | 0.033992 | 2.145116 |
| 4-guanidinobutyric acid | C01035 | 0.5303 | 0.024067 | 2.133159 |
| Vanillin | C00755 | 0.6148 | 0.010673 | 2.058038 |
| Ricinoleic acid | C08365 | 0.5639 | 0.012561 | 1.996042 |
| Trans-vaccenic acid | C08367 | 0.7498 | 0.036669 | 1.906346 |
| 3,4-dihydroxybenzaldehyde | C16700 | 0.757 | 0.039281 | 1.898918 |
| Gamma-aminobutyric acid | C00334 | 0.7525 | 0.034913 | 1.8938 |
| Cis-11-octadecenoic acid | C21944 | 0.7591 | 0.036863 | 1.8929 |
| Quinolinic acid | C03722 | 0.6897 | 0.030964 | 1.5300 |
| Isodeoxycholic acid | C17661 | 0.6244 | 0.008346 | 1.5159 |
| 12-tridecanoic acid | C17076 | 0.6103 | 0.023168 | 1.4375 |
| Lithocholic acid acetate methyl ester | C03990 | 0.7442 | 0.000312 | 1.4032 |
| Taurochenodeoxycholic acid | C05465 | 0.7769 | 0.016839 | 1.2860 |
| 3,7-diketocholanic acid | NA | 0.774 | 0.025305 | 1.1159 |

**Supplementary Table 10** The Correlation between differential metabolites and ADHD symptoms

| Metabolites | Inattention | Hyperactivity | Impulsivity |
| --- | --- | --- | --- |
| Trans-vaccenic acid | -0.156 | -0.210 | -0.109 |
| 3,4-dihydroxybenzaldehyde | -0.161 | -0.211 | -0.113 |
| 12-hydroxystearic acid | -0.348 | -0.309 | -0.190 |
| All-trans-retinoic acid | -0.364 | -0.316 | -0.205 |
| Lithocholic acid acetate methyl ester | -0.300 | -0.297 | -0.262 |
| Cis-11,14,17-eicosatrienoic acid | -0.108 | -0.106 | -0.163 |
| Cis-11-octadecenoic acid | -0.144 | -0.195 | -0.106 |
| Vanillin | -0.343 | -0.283 | -0.408 |
| 4-guanidinobutyric acid | -0.315 | -0.275 | -0.395 |
| Gamma-aminobutyric acid | -0.157 | -0.212 | -0.115 |
| Imidazoleacetic acid | -0.494 | -0.065 | -0.273 |
| 12-tridecanoic acid | -0.310 | -0.194 | -0.215 |
| Taurochenodeoxycholic acid | -0.288 | -0.128 | -0.180 |
| But-2-enoic acid | -0.055 | -0.215 | -0.231 |
| Linoleic acid | -0.032 | -0.291 | -0.221 |
| 3,6-diketocholanic acid ethyl ester | -0.028 | -0.307 | -0.216 |
| Quinolinic acid | -0.214 | -0.287 | -0.320 |
| 3,7-diketocholanic acid | -0.232 | -0.223 | -0.136 |
| Isodeoxycholic acid | -0.225 | -0.197 | -0.203 |
| Ricinoleic acid | -0.232 | -0.232 | -0.343 |
| Trehalose | -0.140 | -0.118 | -0.072 |

**Supplementary Table 11** Analysis of the mediating effect of Imidazoleacetic acid between *Lactobacillus sanfranciscensis* and ADHD symptoms

| Outcomes | Effect | β | SE | 95% Bootstrap CI |
| --- | --- | --- | --- | --- |
| Inattention | Indirect effect | -0.137 | 0.055 | -0.269, -0.048 |
|  | Direct effect | -0.535 | 0.107 | -0.757, -0.315 |
|  | Total effect | -0.672 | 0.124 | -0.932, -0.440 |
| Hyperactivity | Indirect effect | -0.068 | 0.041 | -0.160, 0.015 |
|  | Direct effect | -0.396 | 0.133 | -0.668, -0.132 |
|  | Total effect | -0.464 | 0.130 | -0.737, -0.217 |
| Impulsivity | Indirect effect | -0.100 | 0.071 | -0.281, 0.024 |
|  | Direct effect | -0.318 | 0.126 | -0.526. -0.038 |
|  | Total effect | -0.419 | 0.106 | -0.634, -0.215 |

**Supplementary Table 12** Detailed information of the 16S rRNA sequencing data of mice fecal samples

| sample | Total Pairs Read | Filtered Read | Denoised Read | Merged Read | non-chimeric Read |
| --- | --- | --- | --- | --- | --- |
| WT_1 | 67172 | 62056 | 92.38 | 60886 | 58544 |
| WT_2 | 67240 | 63469 | 94.39 | 58490 | 47506 |
| WT_3 | 67364 | 63192 | 93.81 | 60077 | 48836 |
| WT_4 | 68368 | 63291 | 92.57 | 62212 | 61213 |
| WT_5 | 68628 | 63273 | 92.2 | 62253 | 61192 |
| WT_6 | 68739 | 62357 | 90.72 | 61231 | 59345 |
| WT_7 | 67937 | 62921 | 92.62 | 61971 | 58994 |
| WT_8 | 67975 | 63870 | 93.96 | 60135 | 49315 |
| WT_9 | 67135 | 63090 | 93.97 | 57538 | 46039 |
| ABX_1 | 67512 | 61430 | 90.99 | 60462 | 59269 |
| ABX_2 | 67630 | 62166 | 91.92 | 61462 | 60829 |
| ABX_3 | 67751 | 62376 | 92.07 | 61741 | 61182 |
| ABX_4 | 67653 | 61553 | 90.98 | 60604 | 59862 |
| ABX_5 | 67762 | 59227 | 87.4 | 57972 | 55287 |
| ABX_6 | 68083 | 58334 | 85.68 | 55284 | 42434 |
| ABX_7 | 68219 | 59044 | 86.55 | 57204 | 46025 |
| ABX_8 | 67559 | 57521 | 85.14 | 56063 | 54309 |
| ABX_9 | 67650 | 58455 | 86.41 | 56966 | 54348 |
| ABX_10 | 67758 | 58780 | 86.75 | 56403 | 44768 |
| ABX_11 | 67944 | 58069 | 85.47 | 56447 | 53098 |
| ABX_12 | 67134 | 62923 | 93.73 | 57513 | 45280 |
| ABX_13 | 67852 | 63366 | 93.39 | 60007 | 47440 |
| ABX_14 | 67986 | 63254 | 93.04 | 59912 | 49681 |
| ABX_15 | 68129 | 62769 | 92.13 | 59889 | 48598 |
| ABX_16 | 68236 | 63096 | 92.47 | 60136 | 49789 |
| ABX_17 | 68368 | 63598 | 93.02 | 60197 | 49816 |
| ABX_18 | 68624 | 63637 | 92.73 | 60832 | 51309 |
| FMT-A-R2_1 | 68122 | 62605 | 91.9 | 59229 | 49005 |
| FMT-A-R2_2 | 68365 | 63268 | 92.54 | 60187 | 50318 |
| FMT-A-R2_3 | 68498 | 63656 | 92.93 | 60840 | 49181 |
| FMT-A-R2_4 | 67716 | 62881 | 92.86 | 60017 | 51110 |
| FMT-A-R2_5 | 67739 | 63350 | 93.52 | 60018 | 47614 |
| FMT-A-R2_6 | 67858 | 63321 | 93.31 | 59961 | 49766 |
| FMT-A-R1_1 | 68736 | 62317 | 90.66 | 61251 | 59146 |
| FMT-A-R1_2 | 67912 | 62926 | 92.66 | 62043 | 60611 |
| FMT-A-R1_3 | 67991 | 63335 | 93.15 | 62080 | 60034 |
| FMT-A-R1_4 | 67165 | 62157 | 92.54 | 60526 | 57981 |
| FMT-A-R1_5 | 67266 | 61711 | 91.74 | 58542 | 48954 |
| FMT-A-R1_6 | 67056 | 62587 | 93.34 | 58324 | 45725 |
| FMT-A-C_1 | 67249 | 63048 | 93.75 | 59539 | 48373 |
| FMT-A-C_2 | 67371 | 62581 | 92.89 | 59796 | 51062 |
| FMT-A-C_3 | 67482 | 63014 | 93.38 | 60243 | 50234 |
| FMT-A-C_4 | 67615 | 62997 | 93.17 | 60368 | 51225 |
| FMT-A-C_5 | 67869 | 63188 | 93.1 | 60199 | 48717 |
| FMT-A-C_6 | 67982 | 63880 | 93.97 | 61319 | 51939 |

**Supplementary Table 13** Levene's Test for Equality of Variances and t-test for Equality of Means of KEGG level pathways of mean proportions between groups

| Pathway | Group1 | Group2 | F | Sig. | statistic | pvalue |
| --- | --- | --- | --- | --- | --- | --- |
| Fatty Acid  Biosynthesis | WT | FMT-A-C | 2.173 | 0.164 | 3.100 | 0.008 |
|  | WT | FMT-A-R1 | 3.629 | 0.079 | 0.771 | 0.454 |
|  | WT | FMT-A-R2 | 0.246 | 0.629 | 0.911 | 0.379 |
|  | FMT-A-C | FMT-A-R1 | 1.828 | 0.206 | -5.575 | <0.001 |
|  | FMT-A-C | FMT-A-R2 | 1.332 | 0.275 | -2.505 | 0.031 |
|  | FMT-A-R1 | FMT-A-R2 | 2.926 | 0.118 | 0.352 | 0.732 |
| Biosynthesis of  Unsaturated  Fatty Acids | WT | FMT-A-C | 16.720 | 0.001 | 3.702 | 0.005 |
|  | WT | FMT-A-R1 | 8.138 | 0.014 | -0.519 | 0.614 |
|  | WT | FMT-A-R2 | 11.043 | 0.005 | 1.958 | 0.072 |
|  | FMT-A-C | FMT-A-R1 | 0.785 | 0.396 | -4.012 | 0.002 |
|  | FMT-A-C | FMT-A-R2 | 1.556 | 0.241 | -2.679 | 0.023 |
|  | FMT-A-R1 | FMT-A-R2 | 0.020 | 0.891 | 4.091 | 0.012 |

**Supplementary Figure 1** Species rarefaction curves of ADHD subgroups and group TD

**
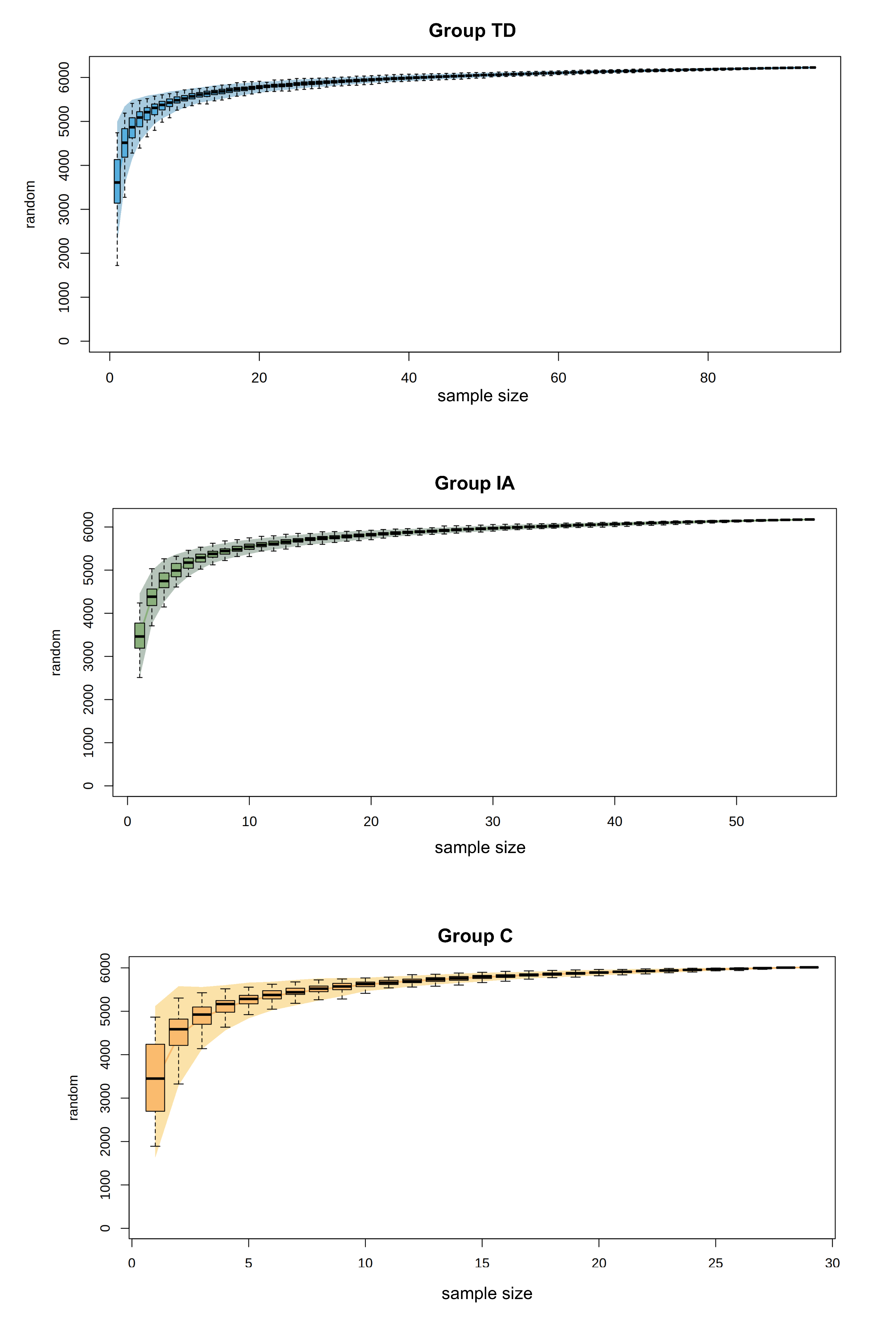
**

**Supplementary Figure 2** The distribution of gene length annotated in all participants

**
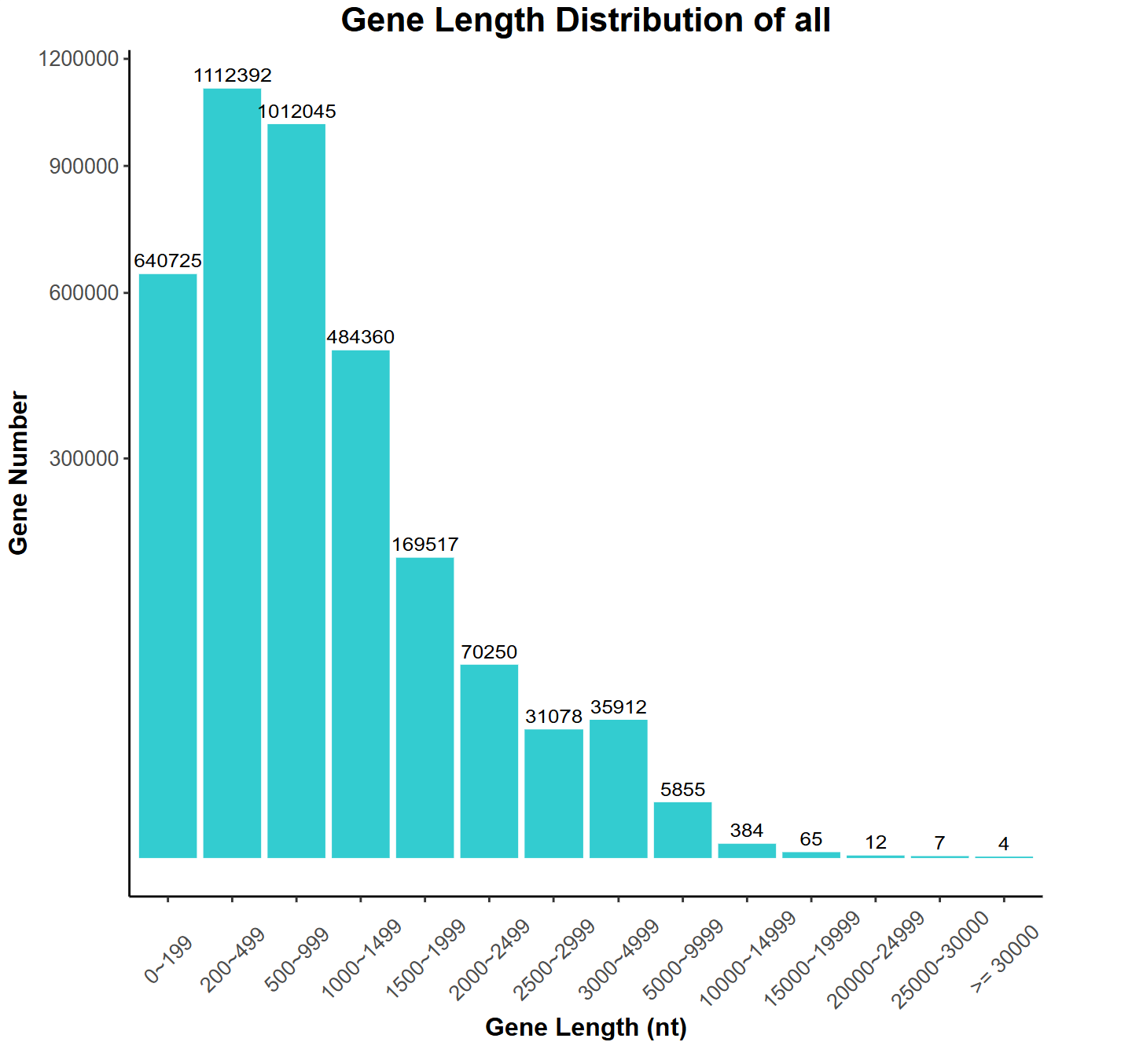
**

**Supplementary Figure 3** The number of genes mapped to Kegg pathways in all participants


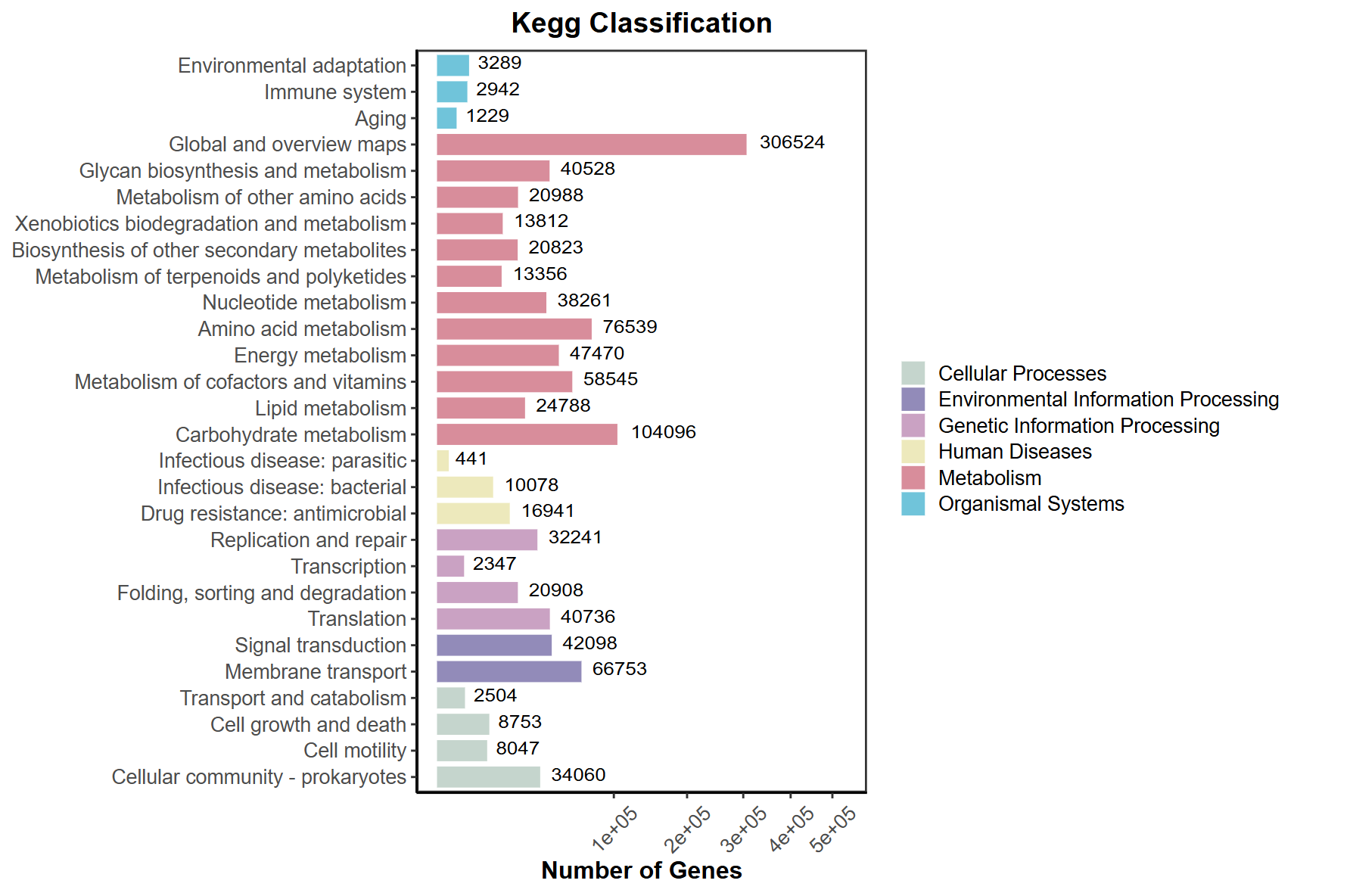


**
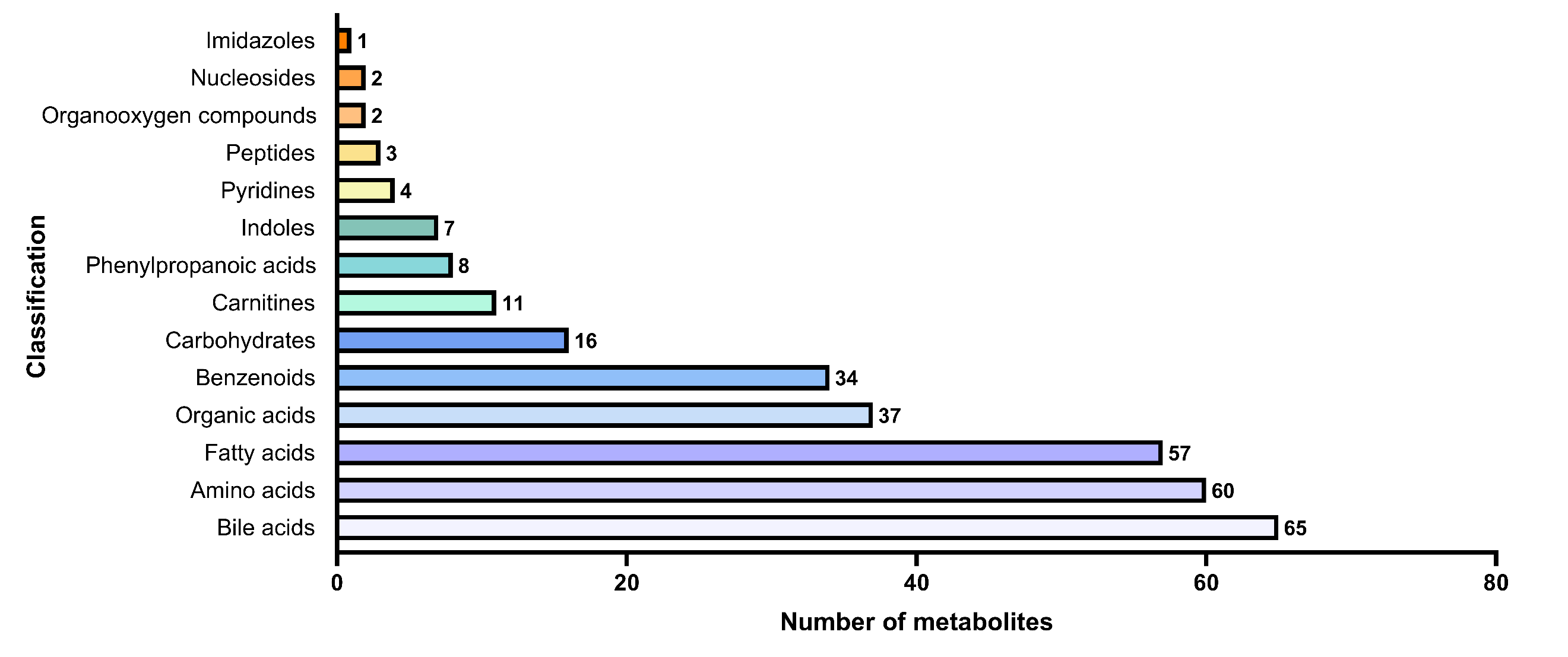
Supplementary Figure 4** The classifications and the number of each class of measured metabolites

**
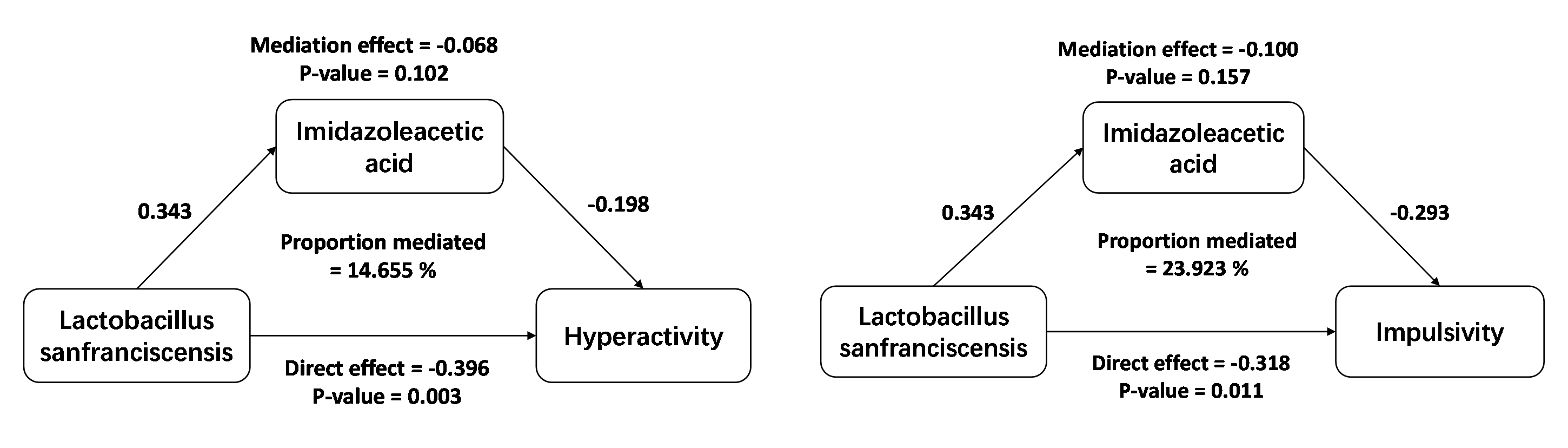
Supplementary Figure 5** Summary plots of causal mediation models of imidazoleacetic acid between *Lactobacillus sanfranciscensis* and ADHD symptoms

**Supplementary Figure 6** The seven successive stages and parameters of 5-Choice Serial Reaction Time Task (5-CSRTT)

**
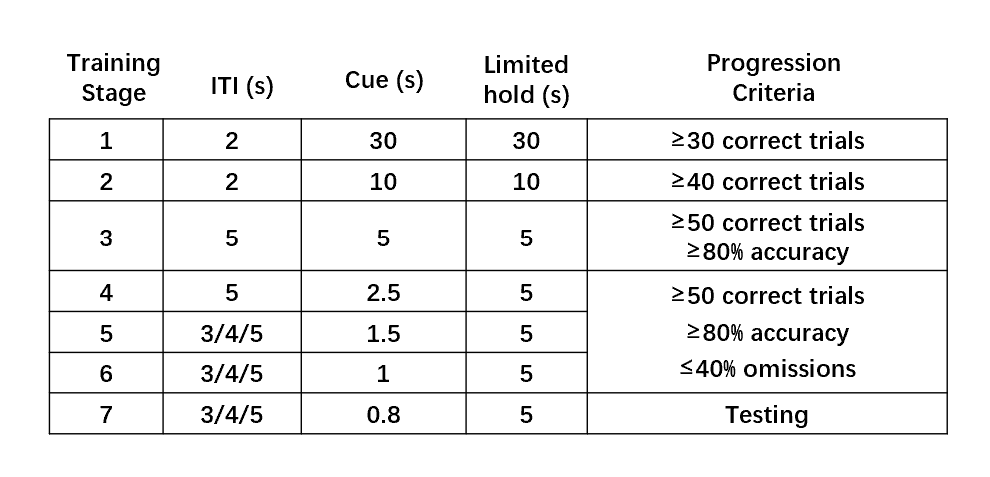
**

**Supplementary Figure 7** The alpha diversity and beta diversity of wildtype and mice after ABX


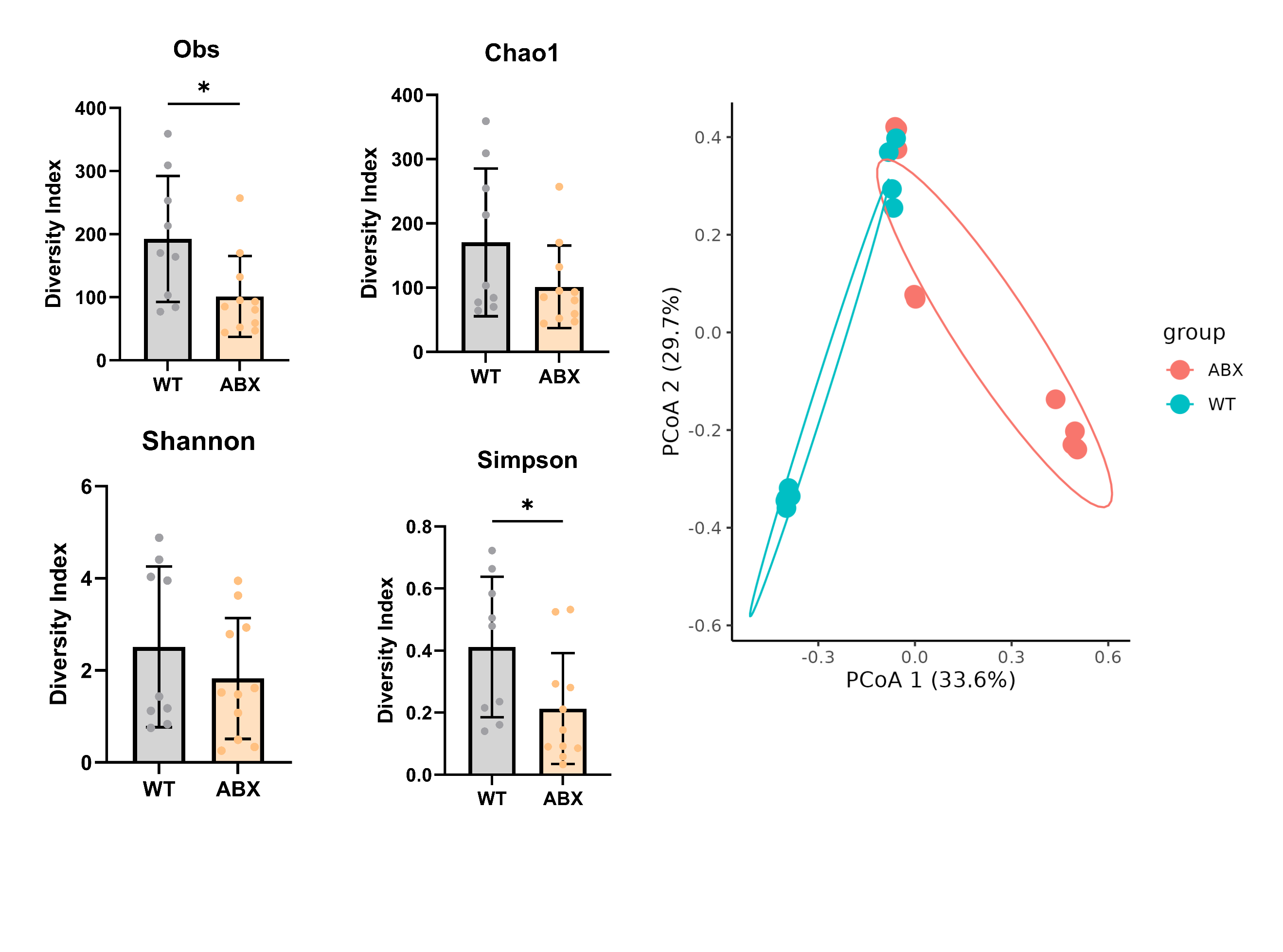


**Supplementary Figure 8** Performance of mice in the open field test and 5-choice serial reaction time task (5-CSRTT) following FMT from different sources

**
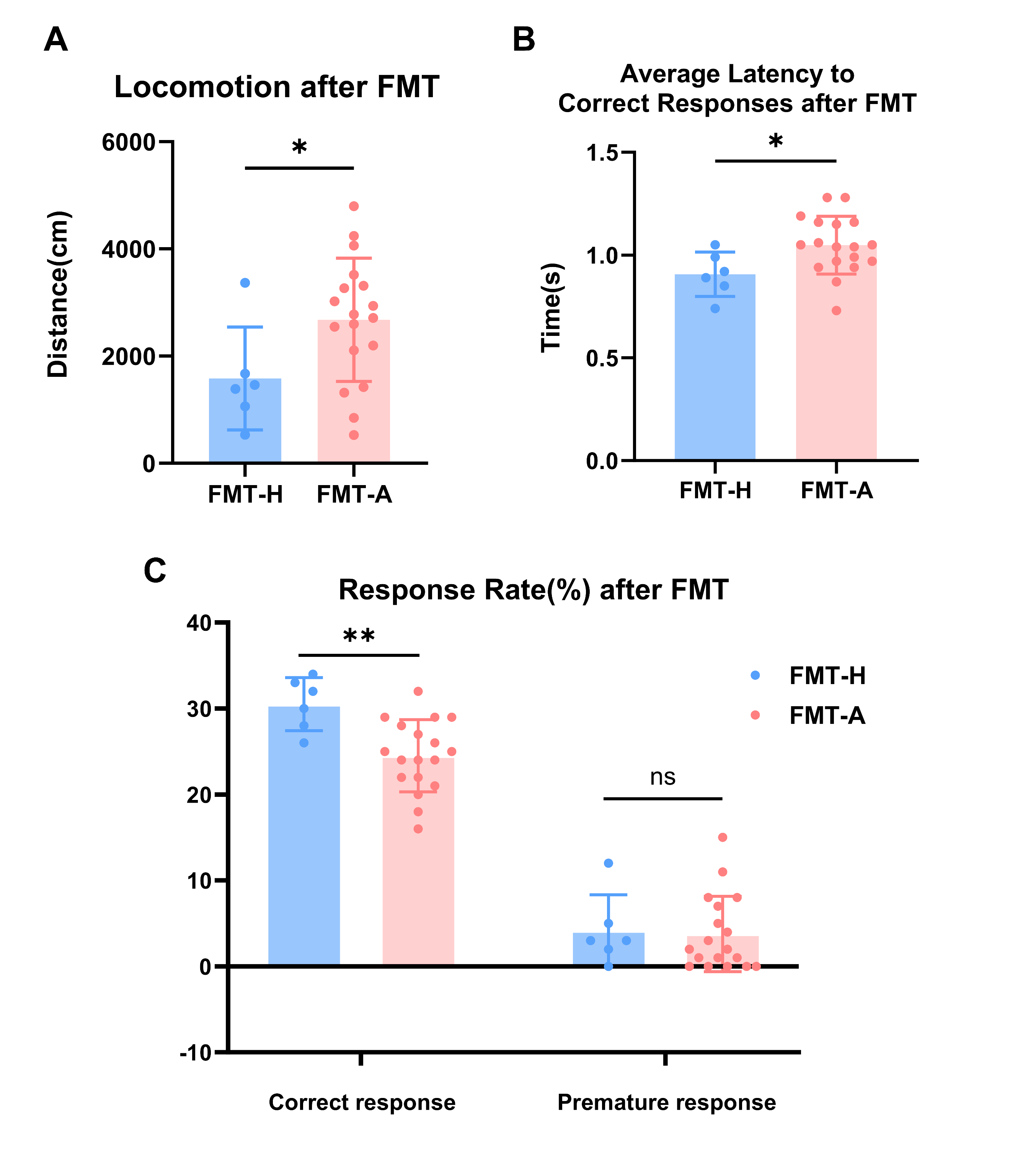
**

**Supplementary Figure 9** The beta diversity of mice following different rescue interventions

**
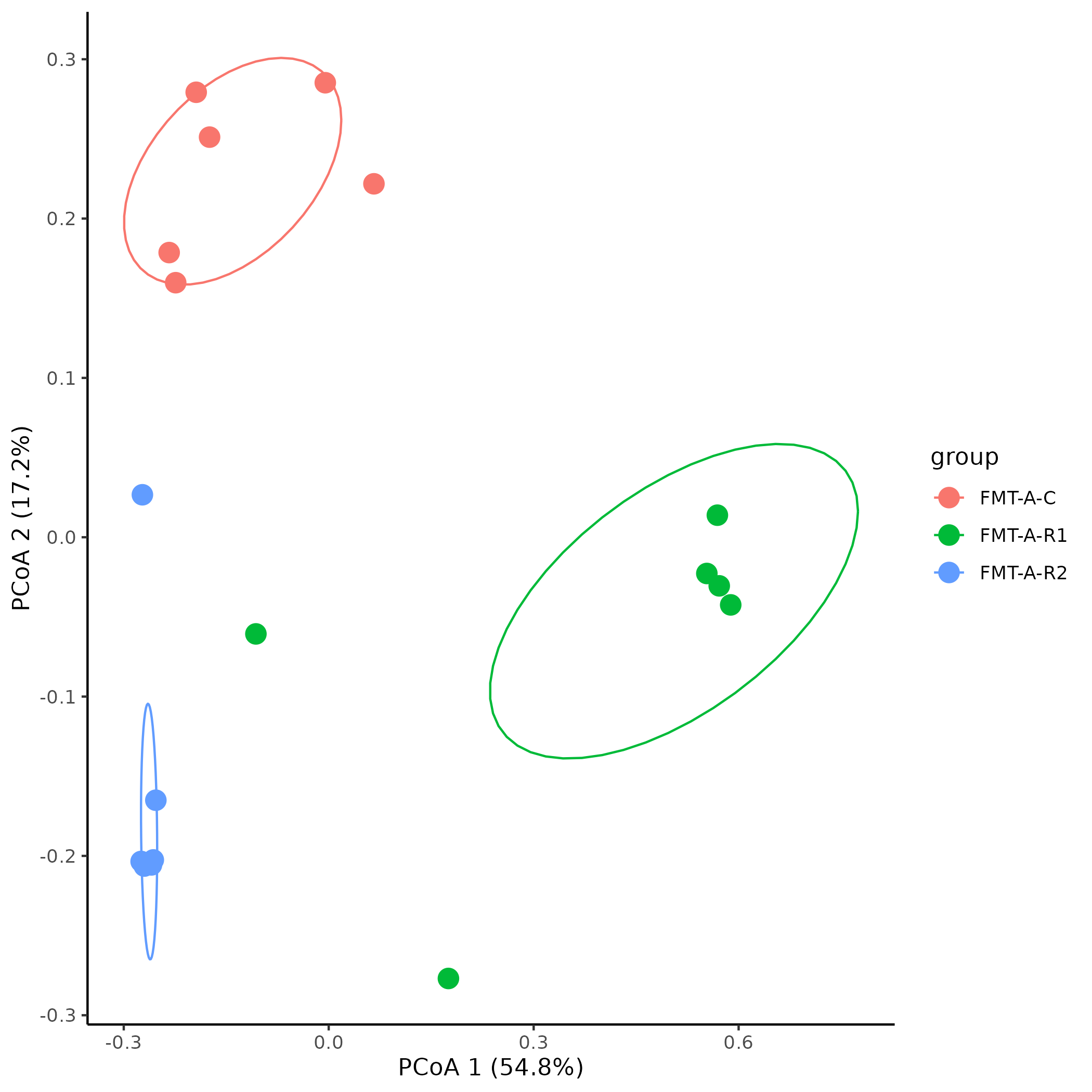
**
